# Overexpression of PSR1 in *Chlamydomonas reinhardtii* induces luxury phosphorus uptake

**DOI:** 10.3389/fpls.2023.1208168

**Published:** 2023-07-27

**Authors:** Stephen P. Slocombe, Tatiana Zúñiga-Burgos, Lili Chu, Payam Mehrshahi, Matthew P. Davey, Alison G. Smith, Miller Alonso Camargo-Valero, Alison Baker

**Affiliations:** ^1^ School of Molecular and Cellular Biology, Centre for Plant Sciences and Astbury Centre for Structural Molecular Biology, Faculty of Biological Sciences, University of Leeds, Leeds, United Kingdom; ^2^ BioResource Systems Research Group, School of Civil Engineering, University of Leeds, Leeds, United Kingdom; ^3^ Department of Plant Sciences, Cambridge University, Cambridge, United Kingdom; ^4^ Departamento de Ingeniería Química, Universidad Nacional de Colombia, Manizales, Colombia

**Keywords:** biomass, micro-algae, polyphosphate, transcription factor, wastewater remediation

## Abstract

Remediation using micro-algae offers an attractive solution to environmental phosphate (PO_4_^3-^) pollution. However, for maximum efficiency, pre-conditioning of algae to induce ‘luxury phosphorus (P) uptake’ is needed. To replicate this process, we targeted the global regulator PSR1 (Myb transcription factor: Phosphate Starvation Response 1) for over-expression in algae. Manipulating a single gene (PSR1) drove uptake of both PO_4_^3-^ and a Mg^2+^ counter-ion leading to increased PolyP granule size, raising P levels 4-fold to 8% dry cell weight, and accelerated removal of PO_4_^3-^ from the medium. Examination of the gene expression profile showed that the P-starvation response was mimicked under P-replete conditions, switching on luxury uptake. Hyper-accumulation of P depended on a feed-forward mechanism, where a small set of ‘Class I’ P-transporter genes were activated despite abundant external PO_4_^3-^ levels. The transporters drove a reduction in external PO_4_^3-^ levels, permitting more genes to be expressed (Class II), leading to more P-uptake. Our data pointed toward a PSR1-independent mechanism for detection of external PO_4_^3-^ which suppressed Class II genes. This model provided a plausible mechanism for P-overplus where prior P-starvation elevates PSR1 and on P-resupply causes luxury P-uptake. This is because the Class I genes, which include P-transporter genes, are not suppressed by the excess PO_4_^3-^. Taken together, these discoveries facilitate a bio-circular approach of recycling nutrients from wastewater back to agriculture.

## 1 Introduction

Unlike other macronutrient cycles, the geochemical phosphorus (P) cycle lacks an atmospheric form for replenishment of soils. Hence increasing demand for fertilizer has depleted mined reserves, while inefficient utilization has led to eutrophication of water bodies from agricultural runoff (Withers et al., 2020). Sources of wastewater (i.e. sewage/industrial output) represent a potential source of P, yet sewage treatment plants (STW’s) rarely recycle it back to agriculture or entirely prevent the pollution of waterways (Elser and Bennett, 2011). Circular bioeconomy solutions are needed, and microalgae have a long history in wastewater treatment (Ridley et al., 2018; Solovchenko et al., 2019; Slocombe et al., 2020). Unlike vascular plants, microalgae accumulate P in Polyphosphate granules (PolyP) which can act as a slow-release fertilizer (Brown and Kornberg, 2004). This allows algal biomass to be employed in this role, returning P to soils in a controlled manner to minimize runoff (McBeath et al., 2007; Baker et al., 2015; Siebers et al., 2019). Algae-based solutions have several problems however, including seasonal or climate growth limitations, and additional land area requirements. Nevertheless, improvements in P-uptake rates and the P content in biomass could increase the efficiency of the remediation process dramatically (Baker et al., 2015; Slocombe et al., 2020).

Under active growth in P-replete conditions, P resources are assimilated into phospholipids and nucleic acids for cell division (Rao et al., 2009; Zachleder et al., 2016). Producing PolyP would be a diversion from these sinks so presumably cellular processes prevent this happening (Zachleder et al., 2016). However, luxury uptake of P can be triggered by restricting other nutrients (e.g. N, S or Zn), resulting in accumulation of PolyP granules in acidocalcisomes (Goodenough et al., 2019). Alternatively, if P-starvation precedes P-resupply then hyper-accumulation, or ‘P-overplus’ occurs upon reintroduction of PO_4_^3-^, and this is part of the Phosphate Starvation Response (Moseley and Grossman, 2009). This response, best understood in *Chlamydomonas reinhardtii*, acts to conserve P, enhance uptake and exploit alternative external P resources (for continued growth) and primes the cell for P hyper-accumulation (Wykoff et al., 1999b). The actual priming phenomenon, which was first recognized in *Micromonas* spp. (Aitchison and Butt, 1973), is understood to be a strategic response to fluctuating supplies of P (Moseley and Grossman, 2009).

Relying on algal PolyP accumulation for wastewater remediation requires stress pre-conditioning and strict control of nutrients; a process that is already constrained to fit into existing wastewater treatment pipelines (e.g. STW’s) (Solovchenko et al., 2019). An alternative is to control the Phosphate Starvation Response (PSR) and induce luxury uptake. PSR includes conservation measures such as replacement of phospholipids with sulfolipids (Bajhaiya et al., 2016) and, through shifts in PO_4_^3-^ importer gene expression profile, increases uptake rate and affinity for PO_4_^3-^ (Shimogawara et al., 1999; Moseley et al., 2006). Additionally, induction of periplasmic phosphatase activity releases PO_4_^3-^ from external organic PO_4_^3-^ sources. Finally, there is increased capacity for PolyP synthesis through upregulation of the PolyP polymerase (*VTC1* etc.) and *VCX1*, a vacuolar Ca-importer (Moseley et al., 2006).

The majority of the above changes are dependent on a single gene: *PSR1* (Phosphate Starvation Response 1). This Myb-type transcription factor was originally identified through the effects of a non-lethal knockout mutant *psr1-1* (Shimogawara et al., 1999; Wykoff et al., 1999b; Moseley et al., 2006; Bajhaiya et al., 2016). The mutant appears to have a complete loss-of-function: see Suppl. info in (Ngan et al., 2015). The *PSR1* gene has global influences, including induction of storage lipid synthesis associated with S-, N- (Ngan et al., 2015) and P-deprivation (Bajhaiya et al., 2016). These last two studies found that over-expression of PSR1 led to starch increases (and expression of associated genes) along with increased cell size (Bajhaiya et al., 2016). High levels of storage lipid in a sub-population of large-celled ‘liporotunds’ was also observed (Ngan et al., 2015). Reports of a transient 5-fold increase in P-content per cell were noted in one of the PSR1 over-expression studies (Bajhaiya et al., 2016) and in a recent study published during submission of our work (Wang et al., 2023).

Our aim in this study was to focus on the effect of PSR1-overexpression on P-uptake in *C. reinhardtii* in terms of gene expression and relate this information to the physiology of enhanced P-uptake and PolyP production. We reveal how P-homeostasis can be manipulated for harnessing the true potential of microalgae for remediation.

## 2 Materials and methods

### 2.1 Algal strains, culture and harvesting

*C. reinhardtii* strain UVM4 (Neupert et al., 2009) and its transformants were cultured in an Algaetron AG230 (Photo Systems Instruments, Czech Republic) in Tris Acetate Phosphate (TAP) media without Na_2_SeO_3_ (Kropat et al., 2011) at 100µmol photons m^-2^ s^-1^ constant light at 25°C. For microscopy, cell lines were grown in bijou containers for 3-4 d without shaking. For the growth experiments in batch culture, triplicate cultures (lines UVM4, 8-27, 8-42 and 8-2) were inoculated from a 3-4 d starter culture for an initial OD_750nm_ of 0.005 in 250 mL conical flasks with foam bungs, shaken at 150 rpm. Therefore, supply of air was through unassisted diffusion alone. Samples (1-30 mL) were centrifuged at 5000 g for 10 min and the supernatants were filtered (0.22 µm pore size Millex-GP Syringe Filter Unit, Merck) and frozen at -20°C for measuring medium composition. The biomass pellets were washed twice with deionized H_2_O, flash frozen with liquid nitrogen and stored at -80°C.

### 2.2 Generation of the pCL8 construct for PSR1-OE

Constitutive over-expression of the PSR1 gene (PSR1-OE) in algae was driven by the pCL8 construct which included the PSAD gene promoter of *C. reinhardtii*, followed by the RBCS intron, to maximize expression. A synthesized genomic fragment suitable for Golden Gate cloning was used for the PSR1-OE construction. Full assembly of the pCL8 construct used for the transformation was as follows. Assembly of the pCL8 construct was carried out using Golden Gate cloning (MoClo Plant Kit, Addgene) (**Supplementary Figures 1–3**) (Crozet et al., 2018). Correct assembly was confirmed by sequencing (Genewiz). Primers and DNA constructs are listed in **Tables S1, 2**. Full cloning history is depicted in **Supplementary Figure 3**. The PSR1 gene (Cre12.g495100.t1.1) with an inserted 3xHA-tag was synthesized (Genscript Biotech Corporation, UK) and cloned into pUC57 via the StuI restriction site. This plasmid was then used as a template for Golden Gate-based cloning (MoClo Plant Kit, Addgene) (Crozet et al., 2018). The following level 0 plasmids were used: pCM0-001 (PSAD prom), pCM0-024 (RBCS2 intron), pCM0-044 (mVenus, incl. Strep-tag), pCM0-114 (PSAD term) all from (Crozet et al., 2018) and L0_PSR1 (PSR1 cloned into pAGM1287 (MoClo Plant Kit) in this study). For the generation of level 2 plasmids for *C. reinhardtii* transformation, the following level 1 plasmids were used: pAGM4673 (L2 backbone, MoClo Plant Kit), pICH41822 (L2 end-linkers MoClo Plant Kit), pICH54011, pICH54022, pICH54033, pICH54044 (Dummies, MoClo Plant Kit), pCM1-27 (ParoR), (Crozet et al., 2018) and L1_PSR1 (PSADprom-RBCS2intr-PSR1-mVenus-PSADterm, this study). All restriction/ligation reactions were performed using BpiI (BbsI) (Fisher Scientific) or BsaI-HF (NEB) together with T4 ligase (NEB) in a total volume of 20 µL containing 1x BSA, 1x T4 ligase buffer, 5U restriction enzyme, 200U T4 ligase. The typical ratio between destination plasmid and entry plasmid/parts was 1:2, using 75 ng of the acceptor plasmid. Level 1 assembly reaction: 20 sec 37°C, 26x (3 min 37°C, 4 min 16°C), 5 min 50°C, 5 min 80°C, hold 16°C. Level 2 assembly reaction: 45x (2 min 37°C, 5 min 16°C), 5 min 50°C, 10 min 80°C, hold 16°C. All plasmid concentrations and quality were determined using a NanoDrop (ND-1000, Labtech). Correct assembly was confirmed by sequencing (Genewiz). Primers and DNA constructs are listed in **Tables S1, 2.**

### 2.3 Generating the PSR1-OE lines

The transgenic lines *C. reinhardtii* 8-27, 8-42 and 8-2 were independent transformants designed to constitutively overexpress the PSR1 gene. UVM4 (Neupert et al., 2009) was transformed by electroporation with the pCL8 construct (described above). For *C. reinhardtii* transformation, UVM4 was grown in liquid TAP for 2 d until mid-logarithmic phase (1-4 x 10^6^ cells/mL). Cells were collected by centrifugation (2500 g, 10 min) and the pellet was washed twice with ice-cold EP buffer (electroporation buffer: 40 mM sucrose, 10 mM mannitol and 10 mM CHES pH 9.25). The pellet was resuspended in EP buffer to a final volume of 1 x 10^8^ cells/mL. Electroporation was performed using a NEPA21 (Nepa Gene Co. ltd.) in 0.2 cm cuvettes (Nepa Gene Co. ltd.) at: 2x poring pulse 300 V, 4 ms length, 50 ms interval, 10% decay rate, polarity +; 1x transfer pulse 20 V, 50 ms length, 50 ms interval, 40% decay rate, polarity +/-. For each transformation, 25 µL cells (2.5 x 10^6^ cells) and 5 µL Plasmid-DNA (500 ng) were used. After transformation, the cells were kept at dim light (2-3 µE) for 16 h and plated on fresh TAP plates containing 10 µg/mL Paramomycin (Sigma). Plates were incubated for 10 d at 30-50 µE constant light. Colonies were picked and sub-cultured weekly, for 3 weeks in liquid TAP + Paromomycin. Surviving colonies were screened by Colony PCR where 100 µL of cell suspension of each colony was collected by centrifugation (15,000 g 1min). Pellets were resuspended in 50 µL 5% Chelex-100 (Sigma). Samples were boiled for 10 min, cooled down on ice, vortexed and centrifuged again. 1 µL supernatant in a total reaction volume of 20 µL was used as template and PCR was performed using Q5 polymerase (NEB). Primers are listed in **Supplementary Table 1**. Stably transformed lines were screened by western blotting (below) for levels of intact fusion protein.

### 2.4 Confocal microscopy

Culture samples of 200 µL were collected and 2 µL of DAPI stain (stock 1 mM; final conc. 10 µM) was added, and samples were incubated in the dark for 4 h. For nuclear targeting, live cell images were captured with a Zeiss LSM880 + Airyscan Inverted Microscope (Carl Zeiss) using a Plan-Apochromat 40x/1.4 Oil DIC M27 objective. Filters were set as follows: Venus Ex. 514 nm, Em. 520-550 nm; DAPI Ex. 405 nm, Em. 420-475 nm (DAPI-DNA) and 535-575 nm (DAPI-polyP). Chlorophyll autofluorescence was captured with the 514 nm laser at 670-720 nm. Visualization of PolyP from the time course experiment was carried out as above with the following differences. After DAPI incubation (as above), samples were fixed with glutaraldehyde: 25% (SIGMA) stock was added at 20µL/mL of culture and incubated for 20 min before being flash frozen with liquid nitrogen and stored at -70°C for later analyses. A Plan-Apochromat 63x/1.4 Oil DIC M27 objective was used (Zeiss LSM880+ Airyscan Upright Microscope, Carl Zeiss). Time course DAPI-polyP images were obtained as 6-8 Z-stacks and further processed as a Z-projection using the software Fiji (Image J) (Schindelin et al., 2012).

### 2.5 Analysis of medium composition and biomass

Filtered supernatant samples were diluted 5-50 fold in dH_2_O and soluble PO_4_^3-^P determined (Koistinen et al., 2017). In this procedure we performed our own calibration curve of 1-24 μM P. Ammonium concentration (NH_4_^+^-N) in filtered media samples was determined using the Hach^®^ cuvette test LCK-304. Samples were diluted 50x-100x, due to interference by components of TAP media. ICMS measurements were made as follows: anion and cation analyses were performed using an ion chromatographer (Metrohm 850 Professional IC), with an 896 Professional Detector. Sterile filtered supernatant samples were diluted between 10-20X. The anion pump injector used 20 µL of the diluted sample and was analyzed with a Metrosep A Supp 5-150/4.0 separation column (flow rate of 0.7 ml/min). The cation pump injector used 10 µL of the diluted sample, which was analyzed with a Metrosep C4-100/4.0 separation column (flow rate of 0.9 ml/min).

For P in biomass, pellets were dried under vacuum using a SpeedVac Plus (SC210A – Thermo Savant Instruments) overnight, and dry weight was determined. A second drying period (overnight) ensured that dry weight data was accurate. The dry pellets were digested with an oxidizing reagent at 100°C for 60 min, using a Hach Lange LT200 Dry Thermostat (Koistinen et al., 2017), diluted 10X-25X and tested as above.

### 2.6 Protein and western blot analysis

Total protein was extracted according to (Wittkopp et al., 2017) and the equivalent of 10 µg of chlorophyll was loaded per lane for SDS-PAGE electrophoresis and western blot analysis. Chlorophyll was determined as below. Tris-Glycine based SDS-gel (Mini-Protean^®^ TGX, Bio-Rad or Novex™ 8%, Thermo Fisher) and transferred onto a PVDF membrane using the Trans-Blot^®^ Turbo™ Transfer System (Bio-Rad). Equal loading and transfer of the proteins was confirmed by brief Ponceau Red staining (0.1% (w/v) in 5% acetic acid, Sigma), washing in water and de-staining with TBST. The membrane was blocked for 1h in 5% (w/v) skimmed dry milk in TBST. Primary anti-GFP antibody (1:5000, ab6556, Abcam) was preincubated overnight with a membrane containing protein extract of UVM4 before adding to 3% milk TBST for incubation overnight at 4°C. The membrane was washed 3x in TBST for 10 min and incubated with the HRP-conjugated secondary anti-Rabbit antibody (1:5000, 111-035-144, Jackson Immuno Research) in 3% milk TBST for 1 h at room temperature. ECL detection was performed using the SuperSignal™ West Pico Chemiluminescent Substrate (Thermo Scientific).

### 2.7 Chlorophyll measurements

Chlorophyll concentration was determined by pelleting 0.1-1mL *C. reinhardtii* cell culture (10,000 g, 10 min) and resuspending the pellet in 1mL 80% acetone in MeOH. After a second centrifugation step for 5 min, absorbance was read at 663.6 nm, 646.6 nm and 750 nm using a spectrophotometer (Jenway 6715UV/Vis, Geneflow) (Wood et al., 2020).

### 2.8 RNA extraction and sequencing

RNA was extracted by grinding frozen algal pellets in liquid nitrogen, followed by extraction with a Qiagen plant RNA mini kit (Qiagen). Subsequent RNA sequencing was by the Next Generation Sequencing Facility (Leeds Institute of Biomedical & Clinical Sci.). RNA quality was checked using a 2100 Bioanalyzer and Expert software (Agilent). 100 ng total RNA of each sample was used to generate a TruSeq stranded RNA Illumina compatible library, from which rRNA was removed using rRNA-specific depletion reagents (Illumina). After size selection and adaptor removal with AMPure beads (Beckman Coulter), library concentrations were determined by qPCR before combining to make an equimolar pool that was sequenced (75bp single end sequencing read HiSeq3000 lane; Agilent; Santa Clara, USA).

### 2.9 RNAseq analysis

RNAseq data was processed using the Galaxy Server (https://usegalaxy.org) except as noted. The reference genome and gene annotation files were obtained from JGI (https://genome.jgi.doe.go). Sequence data were checked for quality: FastQC (https://www.bioinformatics.babraham.ac.uk/projects/fastqc/) before and after trimming using Trimmomatic. Trimmed reads were aligned using Hisat2 to the reference genome file Chlamydomonas_reinhardtii_JGI_v5.5. Reads were counted with FeatureCounts using the gene annotation file Creinhardtii_281_v5.5.gene_exons.gff3.

For relative gene expression data (Fold change: FC) as log_2_ (FC) with associated significance (P-adj values), FeatureCount files for replicate (n=3) cultures were compared for experimental v. controls using DESeq2 (**Supplementary Data 1**). For gene expression levels, counts were converted to RPKM in Microsoft Excel, normalizing to gene length and total reads (**Supplementary Data 1**). For determining RPKM for elements of the transgenic construct (e.g., YFP) or wild-type specific components of the PSR1 gene (e.g., 3’UTR) in the genome, trimmed files (Trimmomatic) were converted to FASTA files (Fastq to Fasta converter). The sequences within were renamed numerically with Rename sequences (numeric counter) and a blast dbase created for each file using Makeblastdb(nucleotide) before carrying out blastn with the appropriate gene fragments to obtain the counts, which were converted to RPKM as above.

### 2.10 Further transcriptomic data analyses

Version 5.6 gene annotation data for *C. reinhardtii* was downloaded from JGI (https://genome.jgi.doe.go) and assigned to the curated transcriptomic data in Microsoft Excel. Time course data was processed as follows: for investigating changes in the control UVM4, relative gene expression data was derived for d6 v d2 (late) and d3 v d2 (early) as above. In each case genes were ranked in Microsoft Excel by the up and down values to obtain four sets of significantly regulated genes (>2-fold P-adj<0.05). Panther gene ontology codes from the annotation were used to analyze gene function for the four gene sets at http://www.pantherdb.org/. In addition, the top 200 ranked genes by FC for each set were manually curated into gene functional roles (and unknowns) which were formulated to match the specific requirements of this study using data supplied on the JGI genome browser (https://genome.jgi.doe.go) for each gene accession (**Supplementary Data 1**).

For investigating the transgenic PSR1-OE lines, FC's were obtained for each line relative to UVM4 for d2, 3 and 6. Here, a list of biologically significant genes (OE-248) was obtained by including those where at least one time point was up or down by >2-fold for at least one transgenic line. In this case, each gene was designated either up or down according to which change had the greatest magnitude. Similar treatment was applied to the P-STRESS dataset (Bajhaiya et al., 2016) (time-points d3 and d5) so the two datasets could be compared by Venn diagram analysis (**Supplementary Data 1**). In the latter case, P-adj values were not available hence the use of the FC cutoff. The OE-248 set was curated into functional roles and processes as above, annotating genes if necessary and considering *C. reinhardtii* biology (**Supplementary Data 1**).

### 2.11 Statistical analysis and data processing

Statistical differences were evaluated by one-way ANOVA and by Tukey HSD test with a p-value of 0.05; using OriginPro (Version 2021, OriginLab Corporation, Northampton, MA, USA).

The growth rates and doubling times for each line were calculated according to (Osundeko et al., 2013). The specific growth rate (*µ = d^-1^*) was calculated for the exponential growth phase as follows: *µ=(ln(y_1_/y_0_))/(t_1_-t_0_)*, where y_1_ and y_0_ correspond to the biomass concentration values at the beginning and at the end of the exponential phase, respectively, and t_1_ and t_0_ are the days where y_1_ and y_0_ were obtained. The doubling time (d) was calculated as *ln(2)/µ*. Biomass productivity (*g L^-1^ d^-1^*) was determined with the equation *Bp= (Bc_f_ - Bc_i_)/t*, where *Bc_i_* and *Bc_f_* are the biomass concentration initial and final values for the cultivation time (t=7 d), respectively. The nutrient uptake and removal rates for each line were calculated according to (Lavrinovičs et al., 2021). The maximum nutrient removal rates R_max_ (mg N L^-1^ d^-1^) were obtained by calculating the daily removal of a specific nutrient (CN_d_ – CN_d-1_) where CN_d_ is the nutrient concentration at a specific day and CN_d-1_ is the nutrient concentration on the day before, and finally selecting the highest removal rate observed on a specific day during the experiment. The nutrient consumption was calculated as *V (mg N g^-1^ dw) = (CN_0_-CN_1_)(Bc_1_-Bc_0_)*, where *CN_0_* and *CN_1_* are the media nutrient concentration values and *Bc_1_* and *Bc_0_* are the biomass concentrations at the early (t*_0_* =d1) and late exponential phase (t*_1_* =d4). The nutrient uptake rate *k* (d^-1^) was obtained by dividing the nutrient consumption by the specific growth rate *µ*.

Multivariate analyses by PCA and Pearson’s correlation coefficient plots were carried out using PAST v4.08 (Hammer et al., 2001). PCA was carried out on mean (n=3) values for log_2_(FC) values (**Supplementary Data 1**) for two transgenic lines (8-27, 8-42) v. UVM4 control (6 data points per gene: 3 time points, 2 transgenic lines). PCA was also carried out on RPKM data (**Supplementary Data 1**) for the above 3 lines, along with PI, PE and N data using data means (n=3) (9 data points per gene: 3 time points, 3 lines). Pearson’s correlation coefficients were also determined from these RPKM data in relation to the PI, PE and N data (**Supplementary Data 1**).

## 3 Results

### 3.1 PolyP accumulation is increased with PSR1 over-expression

**Figure 1:**
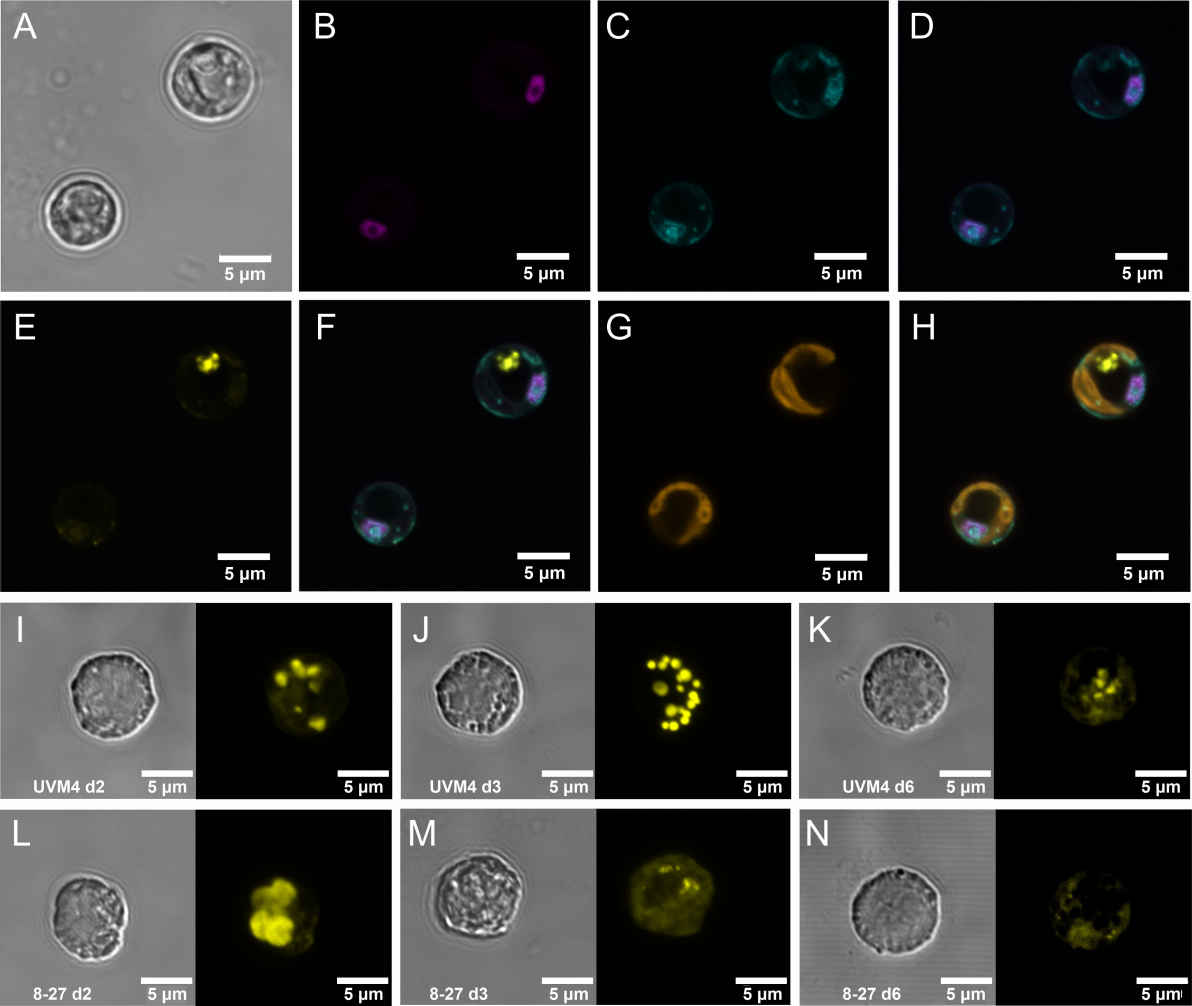
Intracellular localization of PSR1-YFP fusion protein and associated increases in PolyP storage granules. Intracellular localization, as determined by fluorescence confocal microscopy, of the PSR1-YFP fusion protein shown in **(A-H)** for two representative cells from PSR1-OE line 8-27 grown in TAP media. **(A)** bright-field images indicating cell diameter. **(B)** Venus-YFP signal (Emission λ 520-550 nm: magenta) indicating targeting to the nucleus which was identified by DAPI-DNA fluorescence (Emission λ 420-475 nm: cyan) **(C)**, followed by co-localization of the DAPI and YFP signals in the merged image **(D)**. The PolyPhosphate (PolyP) granules are indicated by the DAPI-PolyP (Emission λ 535-575nm: yellow) **(E)**. These are visible as separate entities from the DNA-DAPI stain in the merged image **(F)**. Chlorophyll UV-fluorescence (Emission λ 670-720 nm: orange) indicating the single large cup-shaped chloroplast **(G)** and the merged image **(H)** placing the PolyP signal to the periphery of the dark central region of the cell (vacuole). Displayed in **(I-N)** are differences in the accumulation of PolyP granules in cells from a batch culture time course in TAP media shown in **Figure 2**, comparing Replicate 1’s of the control line UVM4 **(I-K)** and the PSR1-OE line 8-27 **(L-N)** at three different time points (indicated). Each panel is split between bright field (left) and the DAPI-PolyP signal (right) (Emission λ 535-575nm). A representative cell image was taken from multiple cell images shown in **Supplementary Figure 6**.

**Figure 2:**
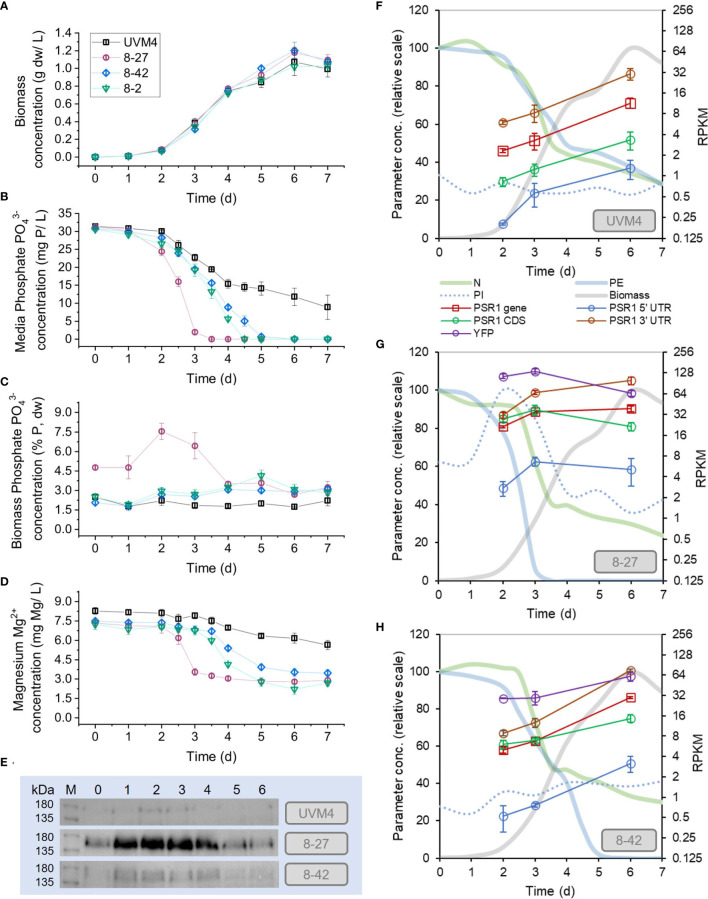
Enhanced phosphate removal and accumulation in PSR1-OE lines. Measurements are shown of medium and biomass composition, along with PSR1 expression levels during batch culture in TAP medium. PSR1-OE lines 8-27, 8-2 and 8-42 with UVM4 control were sampled. **(A)** Biomass dw concentration; **(B)** PO_4_^3-^ in media (PE) and **(C)** PO_4_^3-^ mass concentration in biomass (PI), determined by colorimetric assay; **(D)** Mg^2+^ concentration in media determined by ICMS. **(E)** Anti-YFP antibody western showing PSR1-YFP fusion protein with equal chlorophyll loadings (10 µg), where d0 refers to the starter culture prior to dilution. Complete western analysis shown in **Supplementary Figure 8**, indicating similar protein loadings. **(F-H)** mRNA levels determined by RPKM for PSR1 showing non-specific full mRNA (“gene”) and CDS fragments, along with one transgene-specific YFP fragment and two endogenous gene-specific UTR fragments. Normalized PE, PI and N data shown for comparison. Error bars indicate SE, n=3 culture replicates.

Growth under replete conditions led to transiently enhanced PolyP accumulation and increased granule size with PSR1 over-expression (**Figure 1**). This was achieved by over expression of PSR1 in *C. reinhardtii* strain UVM4 (Neupert et al., 2009) using a constitutive promoter driving a C-terminal PSR1-YFP fusion (predicted size 109.4 kDa, 1048 amino acids) (**Supplementary Figures 1-3**, **Supplementary Tables 1, 2**). Three independently transformed lines (8-27, 8-2 and 8-42) were generated (**Supplementary Figure 4**). The PSR1-YFP fusion was found to be targeted to the nucleus in transgenic lines 8-27 (**Figures 1A–H**) and 8-42 (**Supplementary Figure 5)** by confocal microscopy. We tested all three lines and control under batch culture in TAP medium (1mM P~30 mg/L; 25°C under continuous light) (**Figure 2**). The strongest PSR1-OE line and UVM4 control were examined for PolyP in **Figures 1I–N**, with the full data set comprising 10 cells for each measurement shown in **Supplementary Figure 6**. In UVM4, multiple discrete PolyP granules were visible over the timecourse at ≤1 micron diameter (d2-6). In contrast, line 8-27 showed enlarged PolyP granules (≤3 microns across on d2), and a greater overall PolyP (DAPI) signal compared with the control. This was transient and, beyond d2, PolyP signal became markedly diffuse in 8-27, with a weaker signal and smaller granule size than the control.

### 3.2 Enhanced uptake of phosphate with over-expression of PSR1

We found enhanced P-uptake in all the transgenic PSR1-OE lines relative to the UVM4 control (**Figure 2**) and the strongest line reached biomass P-content of 8% dw P, a 4-fold increase over the control (**Table 1**). No significant growth rate penalties were seen with PSR1-OE, when compared with the untransformed control (**Figure 2A**; **Table 1**). In the case of UVM4, removal of P from the medium was only partial at ~50% after 7d of culture despite reaching stationary phase (**Figure 2B**). Biomass P-content remained unchanged at 2% dw throughout culture (**Figure 2C**).

**Table 1:** Physiological parameters during PSR1 overexpression in batch culture over time (7 d).

Parameter	Units	UVM4	8-27	8-42	8-2
Specific growth rate	d^-1^	1.44 ( ± 0.04)*	1.33 ( ± 0.03)	1.42 ( ± 0.01)	1.42 ( ± 0.02)
Doubling time	d	0.48 ( ± 0.01)	0.52 ( ± 0.01)	0.49 ( ± 0.01)	0.49 ( ± 0.01)
Biomass productivity	g dw L^-1^ d^-1^	0.14 ( ± 0.01)	0.16 ( ± 0.01)	0.15 ( ± 0.01)	0.15 ( ± 0.01)
PO_4_^3-^ uptake rate†	mmol P g dw^-1^d^-1^	0.48 ( ± 0.08)	0.97 ( ± 0.04)	0.65 ( ± 0.02)	0.75 ( ± 0.01)
Mg^2+^ uptake rate	mmol Mg g dw^-1^d^-1^	0.09 ( ± 0.01)	0.33 ( ± 0.03)	0.16 ( ± 0.01)	0.22 ( ± 0.04)
PO_4_^3-^ R_max_	mM P L^-1^ d^-1^	0.23 ( ± 0.05)	0.73 ( ± 0.02)	0.34 ( ± 0.02)	0.44 ( ± 0.07)
Mg^2+^ R_max_	mM Mg L^-1^ d^-1^	0.08 ( ± 0.01)	0.29 ( ± 0.04)	0.12 ( ± 0.03)	0.22 ( ± 0.02)
P/Mg R_max_ Molar ratio	–	2.88	2.52	2.83	2.00
Time of R_max_	d	4	3	4	4
Biomass P_max_	%P dw	2.21 ( ± 0.41)	7.55 ( ± 0.62)	3.11 ( ± 0.16)	4.14 ( ± 0.75)
Time of Biomass P_max_	d	7	2	7	5

*S.E. of mean (n=3). †Uptake rate calculated for log phase which was taken as d1-4.

In contrast, all three PSR1-OE lines showed complete removal of PO_4_^3-^ by 5d, before the point of zero growth, displaying significantly higher P-content in biomass during most of batch culture than UVM4 (**Figures 2B**, **C**; statistical tests in **Supplementary Figure 7**). In the strongest line (8-27), complete removal of PO_4_^3-^ was seen earlier at 3-4 d of growth, and low biomass concentration (~0.4 g dw/L). Here, maximum P-uptake rate (R_MAX_) was 3-fold higher than that of UVM4, compared with 1.5-2-fold for lines 8-2 and 8-42 (**Table 1**). Temporal differences in peak P-content were noted between lines. In line 8-27 P-content peaked at d2 (8% dw P) with a lesser peak at d5 for the next best uptake line, 8-2 at 4% (**Figure 2C**; **Table 1**).

### 3.3 Phosphate hyperaccumulation linked to magnesium uptake

We placed P-hyperaccumulation into context by measuring key inorganic ions in the medium alongside the PO_4_^3-^ measurements (**Figure 2D**; **Supplementary Figure 8**). Interestingly, differing P-uptake rates across the various lines (**Figure 2C**) were closely mirrored by Mg^2+^ losses from the medium over time (**Figure 2D**). Furthermore, calculated uptake rates (R_MAX_) exhibited a P/Mg molar ratio of 2-3 across all four lines (**Table 1**). Uptake rates of other inorganic ions or pH were not altered between the transgenic lines or control: it was only PO_4_^3-^ and Mg^2+^ where this was seen.

Sigmoidal increases in biomass concentration over time in UVM4 background control gave associated decreases in certain ions from the culture medium and an increase in culture pH. For instance, by d7, removal of both PO_4_^3-^ and NH_4_^+^ from growth media was down to 30% in the control. Lesser decreases were seen with SO_4_^2-^ down to 50% and Mg^2+^ down to 80% whereas, K^+^ and Ca^2+^ levels showed little change (**Figures 2B, D**; **Supplementary Figure 8**).

### 3.4 Positive autoregulation mechanism revealed for PSR1

To examine gene expression, RNA sequencing was performed on time points (d2, 3, 6) from the batch culture experiment in **Figure 2** for lines 8-27, 8-42 and UVM4 (**Supplementary Data 1**), and western blotting was also carried out for all timepoints. Unexpectedly, overexpression of the PSR1-YFP transgene also increased expression of the endogenous PSR1 gene (in the examined lines 8-27 and 8-42 relative to UVM4 control). Therefore, the early peak in P-uptake rate in the strongest line (8-27) appeared to be driven by an early rise in both the PSR1-YFP transgene and the endogenous gene.

PSR1-YFP fusion and endogenous PSR1 gene mRNA levels were quantified along with PSR1-YFP fusion protein levels in **Figures 2E–H**. The endogenous gene expression was monitored by measurement of wild-type specific 5’UTR and 3’UTR fragments. The transgene was monitored using its specific YFP fragment, and the fusion protein by using a YFP-specific antibody. For comparison, the complete PSR1 gene mRNA fragment (5’UTR, CDS and 3’UTR) is shown, which was a combination of wild-type and transgene signals.

The strength of the P-uptake response across the lines (8-27, 8-42, UVM4) (**Figures 2B, C**) was proportionate to both total PSR1 mRNA levels and PSR1-YFP protein (**Figures 2E–H**). The early P-uptake response of the strongest line (8-27) associated with early increases in both the endogenous PSR1 (10-fold over UVM4 at d3) and the transgene, along with substantial increases in the PSR1-YFP fusion protein from d0-d2. In contrast, the later P-uptake response of the weaker line (8-42) was associated with a late increase of PSR1 mRNA and lower levels of the PSR1-YFP fusion. Generally, YFP antibody signal matched the transgene mRNA (YFP) indicating dependence. Measurements of total PSR1 mRNA changes by FC (**Table 2**) agreed with the RPKM data (**Figures 2F–H**). For instance, 10-fold for 8-27 over UVM4 at d3, which decreased to 3 for d6. This compared with a constant 3-fold increase for 8-42 v. UVM4 over time (p-adj <0.05).

**Table 2:** Key genes with roles linked to P-homeostasis.

Gene	Accession	Class	Description	Correlation	Fold-change (FC)					
					Line 8-27			Line 8-42		
P-transport				PSR1	d2	d3	d6	d2	d3	d6
PTB3	Cre07.g325740	I (ABCD)	Na^+^/Pi symporter	0.81*	**26.4**	**26.7**	**8.4**	**4.9**	**2.8**	**3.2**
PTB2	Cre07.g325741	I (ABCD)	Na^+^/Pi symporter	**0.82**	**8.7**	**11.6**	**9.0**	**2.7**	2.0	1.7
PTB4	Cre02.g144750	I (ABCD)	Na^+^/Pi symporter	**0.80**	**9.9**	**7.2**	**8.1**	**2.2**	1.5	1.9
PSTS	Cre01.g044300	II (ACD)	ABC transporter	**0.83**	**2.8**	**7.9**	**4.6**	1.3	1.2	1.3
PTB12	Cre02.g144650	II (ACD)	Na^+^/Pi symporter	**0.79**	1.1	**7.0**	**3.0**	0.9	0.9	0.6
PTB6	Cre16.g655200	II (A)	Na^+^/Pi symporter	0.07	**4.4**	0.9	0.9	1.1	0.9	1.0
MFT22	Cre07.g354150	II (ACD)	Major facilitator permease	**0.96**	**3.0**	**3.9**	**2.7**	1.5	1.2	1.7
PTA4	Cre16.g686850	II (A)	H^+^/P symporter	0.28	**3.7**	2.1	1.2	1.1	1.1	0.86
PTA1	Cre02.g075050	I (ABCD)	H^+^/Pi symporter	-0.17	**2.9**	0.8	0.5	**2.0**	1.5	0.7
PTB5	Cre02.g144700	II (ACD)	Na^+^/Pi symporter	**0.73**	1.3	**2.6**	**2.6**	0.9	0.9	1.1
PTA3	Cre16.g686750	I (ABC)	H^+^/Pi symporter	-0.23	**2.1**	1.4	1.7	1.6	1.2	**0.37**
ERM7	Cre12.g532151	III (B)	Ca-dependent channel	-0.02	0.60	0.65	0.72	**0.29**	**0.32**	**0.30**
P-salvage/sparing										
PHO5	Cre04.g216700	II (ACD)	Exophosphatase	0.47	**11.2**	**95.7**	2.0	0.9	1.2	1.2
GDP2	Cre16.g683900	II (ACD)	PLC-like phosphodiesterase	0.47	**11.0**	**76.6**	**4.2**	N/A	1.0	0.9
MPA2	Cre09.g404900	II (ACD)	Metallo phosphatase	0.50	**18.6**	**20.7**	**7.7**	1.6	1.2	0.9
PHO1	Cre08.g359300	II (ACD)	Exophosphatase	0.54	**14.7**	**11.9**	**6.1**	1.2	1.3	1.3
PWR12	Cre16.g693819	II (ACD)	PD-(D/E)XK nuclease	**0.77**	1.5	**2.8**	**5.9**	1.4	1.6	1.6
EEP2	Cre06.g295100	II (AD)	Nuclease/phosphatase	0.41	**4.4**	**5.7**	**3.6**	0.8	0.8	1.2
Phospho1	Cre02.g114500	II (ACD)	Phospholipid phosphatase	**0.93**	**3.7**	**5.2**	**2.7**	1.7	1.3	1.6
SPD2†	Cre04.g218200	II (AC)	Sphingomyelin phosphodiesterase	**0.83**	1.5	**4.2**	**3.2**	0.9	1.2	0.8
SQD3	Cre16.g689150	II (ACD)	Sulpholipid synthase	**0.79**	1.1	**2.7**	**3.2**	1.0	1.0	1.2
PHT1	Cre08.g364100	II (ACD)	Phytase	0.35	1.7	**3.3**	0.9	0.7	1.3	0.9
P-homeostasis										
PSR1‡	Cre12.g495100	I (ABCD)	Global regulator Myb	**1.00**	**11.6**	**11.1**	**3.3**	**3.1**	**3.0**	**2.7**
SPX1†	Cre07.g325950	II (ACD)	Soluble SPX-protein	**0.89**	1.7	**3.2**	1.8	1.4	1.4	1.3
PolyP synthesis										
PLH1	Cre02.g078400	II (ACD)	P-loop NTP hydrolase	0.56	**4.6**	**5.7**	1.4	1.5	0.8	1.4
CAX1	Cre12.g519500	II (ACD)	Ca^2+^/H^+^ antiporter	**0.73**	**3.2**	**3.1**	1.9	1.5	1.3	0.7
VTC4	Cre09.g402775	II (ACD)	PolyP polymerase catalysis	**0.72**	1.7	**2.4**	1.7	1.4	1.3	1.4
VTC1L	Cre09.g402812	II (ACD)	PolyP polymerase subunit	**0.90**	**1.7**	**2.4**	2.1	1.2	1.3	1.5
Cation transport										
MTP4	Cre03.g160550	II (ACD)	CDF Mn transporter	0.53	**2.5**	**34.9**	**4.5**	0.8	0.7	0.7
CIT1	Cre12.g512950	II (ACD)	Chromate ion transporter	**0.90**	**2.6**	**5.0**	**2.9**	1.5	1.3	1.6
CIT2‡	Cre12.g507333	II (ACD)	Chromate ion transporter	**0.89**	**2.7**	**4.8**	2.2	1.6	1.2	1.2
ACA3	Cre06.g263950	II (ACD)	Na^+^/K^+^-exchanging ATPase	**0.82**	**3.4**	**4.6**	**2.7**	1.6	1.6	1.2
CPR1	Cre02.g144550	II (A)	Na^+^/H^+^ exchanger 5-related	**0.77**	0.9	**2.8**	**3.0**	0.8	1.1	1.47
MTP3‡	Cre03.g160750	II (ACD)	CDF Mn transporter	**0.90**	1.5	**2.1**	2.0	1.4	1.2	1.4

*Significant values in bold. Pearson’s correlation coefficients (RPKM, p<0.05, n=3). FC values in relation to UVM4 control (p-adj<0.05, n=3). In the *psr1-1* mutant P-STRESS experiment: †Ectopic down; ‡Independent up (see **Supplementary Data 1**). Gene class is defined as I-IV where Venn class is indicated in parentheses.N/A, not applicable/available.

### 3.5 The Phosphate starvation response was relatively weak in the control

**Figure 3:**
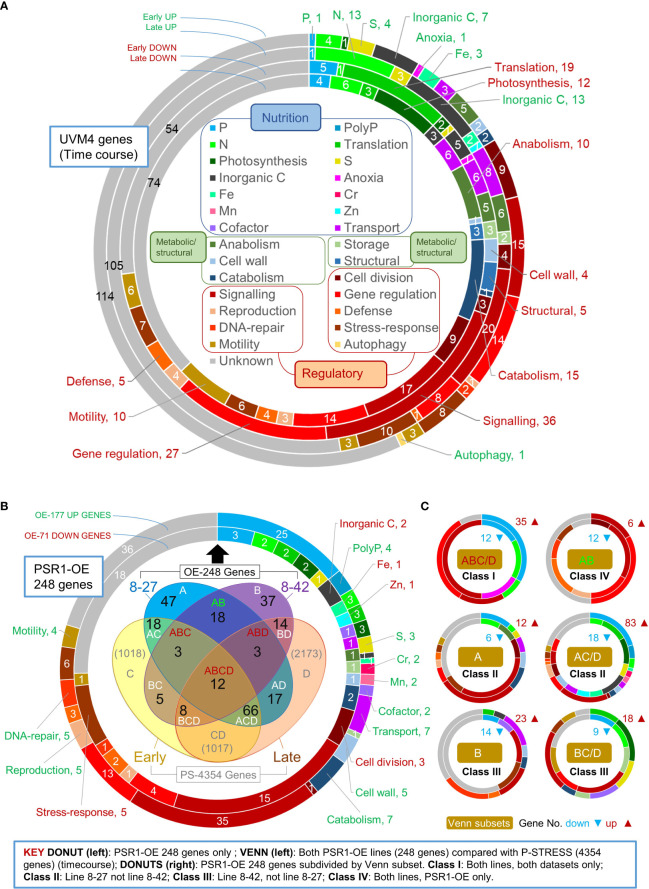
Gene function analysis of transcriptome data. Shown in the upper donut chart **(A)** are relative gene changes (FC) occurring within the background strain UVM4 during batch culture, comparing early (d 3 v. d 2) and late (d 6 v. d 2) for up or down changes. Here gene functional roles are shown for the top 200 expression changes in each up/down category (inset). **(B)** Shown is an analysis of the FC magnitude expression data for 248 genes (OE-248) having a biologically significant change (>2-fold cutoff) in one or more transgenic lines relative to the UVM4 control. The large donut chart (left) shows the gene functional analysis for OE-248 for the up (177) or down (71) regulated genes. The Venn diagram (inset) shows a comparison of OE-248 with PS-4354 comprising 4354 genes obtained from the P-STRESS dataset (>2-fold FC cutoff) for “early” (d3) and “late” (d5). Gene number per Venn sector is indicated (in black for OE-248) and each sector is labeled as follows: A (8-27), B (8-42), C (P-STRESS d3) and D (P-STRESS d5). **(C)** Series of donut charts showing gene functional analysis for six Venn sector subsets of the OE-248 genes, pooling C and D into C/D. These are labeled Class I-IV according to the key (inset). Gene numbers and direction of regulation are indicated (
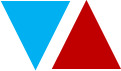
).

Under batch culture on P-replete medium (TAP) there is a backdrop of growth limitations and stresses that develop as biomass increases toward stationary phase. However, gene inductions associated with P-stress were found to be much less evident than for other stresses (**Figure 3A**).

To examine gene expression, RNA sequencing data was analyzed (from time points d2, 3, 6, of line 8-27, 8-42 and UVM4 cultures shown in **Figure 2**; **Supplementary Data 1**). The expression level of a given gene was obtained by measuring its mRNA counts by RNA-seq as a function of the total mRNA population and expressing them as RPKM (Reads per Kilobase Million). To obtain relative changes in gene expression, mRNA counts were compared between two conditions or two time points to obtain the Fold-Change (FC) values. So, large FC values were indicative of a gene that was highly regulated by a condition relative to the control, or by the passage of time. By ranking genes by FC value (**Supplementary Data 1**) it was possible to identify the most important changes in gene expression. Lists of the important genes were drawn up by employing FC ranking cut-offs based on actual or biological significance (see Methods). In **Figure 3**, the gene functional data of these gene lists are depicted using pie-chart donuts. The gene lists are also compared between two different P-homeostasis experiments to identify the commonly regulated genes using a Venn diagram.

In **Figure 3A**, analysis focused on the UVM4 control as it transitions from log-phase to early stationary phase. Because the list was extensive, this was done for the top 200 up- or down-regulated genes ranked by gene expression change, listed in **Supplementary Data 1** (the full list was examined in **Supplementary Figure 9**). Apart from the unknown genes, the largest functional group showing these changes were either of regulatory function or had roles assigned to them in sexual reproduction, motility, or stress responses (30-40%). Genes linked to nutrient limitation or acquisition amounted to 20-30% of the total. Those genes classed as metabolic enzymes or cell structure-related were under 20% of the total.

The impact of a particular stress can be estimated from examining expression changes in genes that are functionally associated with it (e.g., by evaluating gene numbers, magnitude, or early timing). By these criteria, responses to Fe, C, N and S limitation in UVM4 were apparently much greater than those for PO_4_^3-^. For instance, early-induction gene numbers were C (7), S (4), N (4), Fe (3) and P (1) in **Figure 3A**. Genes that showed strong expression changes for early-induction were Fe (FEA1), S (SLT3), or C (CAH8), whereas there was only strong late-inductions for N (NIT3, NAR1.2, and GLN3) or P (PTB12) (**Supplementary Data 1**). Early gene-induction responses potentially attributable to N or P limitation were AAH1 (amino acid catabolism) and GDP7 (a phosphodiesterase, the only early up-regulated P gene) (**Supplementary Data 1**). A few down-regulated early response genes (5) were evident for P including PTA3 (P-transport) and a HAD1 (P-hydrolase) (**Supplementary Data 1**). Early down-regulation of many protein translation genes (19) were followed by late reductions in photosynthetic (12) and metabolic genes (25) (**Figure 3A**; **Supplementary Figure 9**).

### 3.6 PSR1 over-expression mimicked P-stress gene induction not repression

The key question was whether gene regulation patterns associated with increased P-uptake by PSR1 over-expression (under replete conditions) resembled those of P-starvation (as described in the literature). We found that in terms of replicating the P-stress response, PSR1 over-expression was more effective at driving gene induction rather than gene repression.

To address the above key question, our RNA-seq data were compared with a published dataset from a P-starvation experiment (P-STRESS) (**Supplementary Data 1**) (Bajhaiya et al., 2016). The two experiments differed in design where PSR1 over-expression reflected the evolving changes in the culture during growth whereas in P-STRESS, cells were transferred from P replete to P deficient media at a specific time point. Each approach has advantages and disadvantages, but a comparison was essential to validate the behavior of individual genes or their groups.

In **Figure 3B**, the Venn diagram compares the two above P-homeostasis experiments, indicating genes that were significantly regulated in both experiments. The pie-chart donut highlights the functional breakdown of significantly regulated genes from our PSR1-overexpression experiment only. Further Venn diagrams shown in **Supplementary Figure 10,** depict a breakdown of data according to up- or down-regulated genes.

Both P-homeostasis experiments were time-courses therefore, the fold-change (FC) magnitudes were compared in a Venn diagram with a biological significance cutoff of 2-fold (**Figure 3B**). This cutoff generated a subset of 248 genes (OE-248) for our data (98% being significant by Bonferroni correction criteria: p-adj<0.1; 68%, p-adj<0.05; **Supplementary Data 1**) whereas a subset of ~4k was generated with the P-STRESS data (PS-4354 in **Figure 3B**) (**Supplementary Data 1**). It is important to note that the OE-248 set was a pool of all the significantly affected genes in at least one of the two PSR1-OE lines examined relative to the control. The OE-248 data set is listed in **Supplementary Data 1** ranked by FC with key genes shown in **Tables 1**, **2**.

Good agreement was seen between the PSR1-OE and P-STRESS data sets: 60% of the PSR1-OE set was also altered in the P-STRESS set (**Figure 3B**). A higher proportion of PSR1-OE genes were also present in the P-STRESS dataset for the strongest line 8-27, (48% cf. 18% in 8-42). There was relatively low agreement between the two transgenic lines at only 15% of the OE-248 genes (**Figure 3B**). Neither PSR1-OE line showed a bias in altered gene expression toward early- or late-expressed P-STRESS genes (**Figure 3B**). A bias was seen toward gene upregulation in the OE-248 dataset (71%) compared with downregulation (29%), but not within the P-STRESS dataset (**Supplementary Figures 10A,B**). Upregulated PSR1-OE genes were more likely to be up in the P-STRESS data set (55%) than downregulated PSR1-OE genes were down in P-STRESS (27%). These findings also held when a smaller set of highly expressed genes from the P-STRESS dataset were compared to the OE-248 genes, for numerical equivalence (**Supplementary Figures 10C, D**).

### 3.7 Genes affected by PSR1 over-expression were regulated differently by function

**Figure 4:**
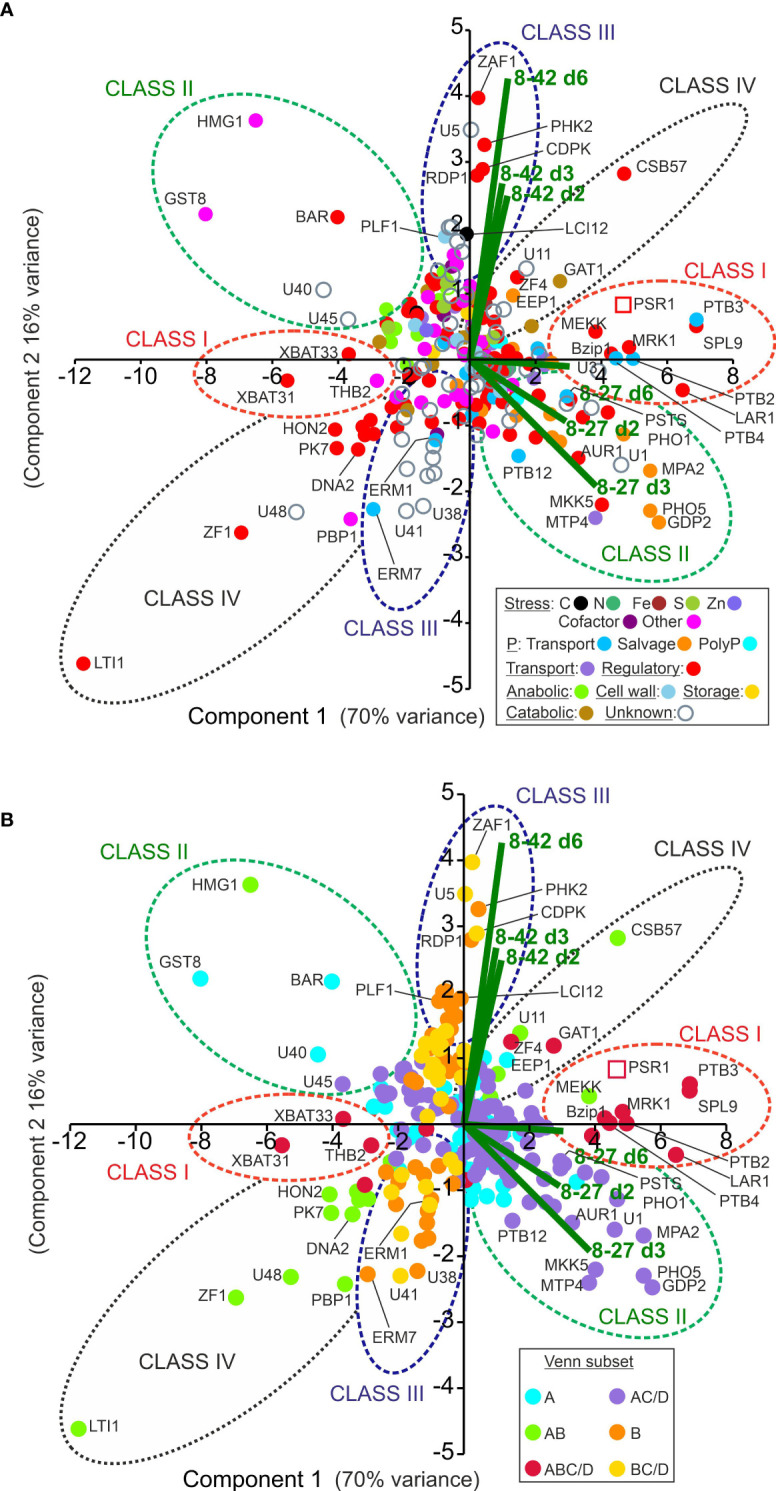
Multivariate analysis of PSR1-OE relative gene expression data. Shown are patterns of gene expression for the OE-248 gene set (significantly regulated genes in line 8-27 or 8-42 relative to UVM4 with >2-fold cutoff). **(A)** Data points are color-coded for the 17 gene functional processes shown (inset). **(B)** The same chart is coded instead for the six Venn diagram sector subsets (inset) as described in **Figure 3B**. In **(A, B)** the mean (n=3) relative gene expression time course data for the two transgenics v. UVM4 (log_2_(FC: fold-change) for d2, 3 and 6; i.e., 6 data points per gene) were analyzed by PCA. Biplots for each of these six data points are shown (–) and key gene expression changes are labeled. Clusters of genes are encircled and labeled Class I-IV. The data point for the complete PSR1 gene mRNA (not specific to endogenous gene or transgene) is shown (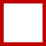).

Comparing the OE-248 data set with the published P-STRESS data through Venn and gene function analysis (**Figures 3B, C**) followed by multivariate analysis (**Figure 4**), placed the genes into four classes having different patterns of gene regulation and function.

Overall, regulatory genes dominated in a functional analysis of OE-248 at nearly 50% (either induced or repressed) (see Donut in **Figure 3B**). Nutrient assimilation/partitioning genes accounted for one third of the changes, where half of the induced genes related to P-stress. Unknowns comprised about 25% and metabolic/structural genes had minor representation.

In **Figure 3C**, gene function pie-chart donuts were generated for Venn diagram subsets depicted in **Figure 3B**. These showed a striking variation in gene function profile between the Venn classes. The six Venn subsets in **Figure 3C** were pools of the twelve OE-248 Venn subsets in **Figure 3B** (made by removing the distinction between early or late in relation to behavior in the P-STRESS dataset: C/D). Further pooling into four classes was possible, considering similarities in gene function profile. For instance, Class II comprised genes that were specific to line 8-27, pooling Venn subsets A and AC/D, because upregulated genes for P-stress were found in both subsets. Likewise, Class III was line 8-42 specific, pooling B and BC/D because down-regulated genes for P-stress were noted in both. In contrast, Venn subsets specific for both PSR1 over-expression lines showed functional differences: Class I (ABC/D, many P-regulated genes) and Class IV (AB: no P-regulated genes) so they were kept separate (**Figure 3C** **)**. We found that subdivision of genes into Classes I-IV were further strengthened by taking into account the strength of gene expression changes, below.

These observations were investigated further by multivariate analysis in **Figure 4** where the complete time course FC data was used instead of the magnitudes, as for Venn. This data-separation analysis identified the key gene expression changes in terms of (i) their differential expression strength (i.e., distance from origin); (ii) induction or repression (i.e., PC1, *x*-axis where approximately, induction *x*>0; repression x<0) and (iii) transgenic line (i.e., PC2, *y*-axis where data bi-plots cluster according to the algal line).

The data were color-coded in **Figure 4A** according to gene function processes or in **Figure 4B** by the six Venn diagram subsets (defined in **Figure 3C**). The genes were found to cluster according to gene function particularly differentiating P-transport (mid-blue) from P-salvage genes (orange) (**Figure 4A**). In **Figure 4B**, the genes fell into four clusters largely conforming to Venn class. There were a handful of exceptions (e.g., GAT1, MEKK and HMG1) that could now be more accurately reassigned to Class I-IV according to the PCA analysis (**Figure 4B**).

The findings for the four classes are summarized as follows (Venn classes: A (line 8-27), B (line 8-42), C (P-STRESS early) and D (P-STRESS late):

Class I genes (Venn set ABC/D in **Figure 3C**) comprised a small but robustly substantiated group of 18 genes that were differentially expressed in both transgenic lines (A, B) and the P-STRESS dataset: early (C) or late (D) or both). It was mostly P-transport genes (e.g., PTB 2-4, PTA1, 3 in **Table 2**) and regulatory genes (e.g., SPL9, Bzip1, MRK1, MEKK and XBAT31 in **Table 3**) in this grouping. The exception was a strongly up-regulated peptidase, GAT1 (**Table 3**). MEKK, a novel early induced regulatory gene (**Table 3**), was re-assigned to Class I from the Venn AB set based on the PCA (**Figure 4B**). PTB2,3 and SPL9 were highly upregulated in both lines, being in the top 20 ranked by FC, in both cases (**Supplementary Data 1**).

**Table 3:** Key genes with roles unassigned or linked to processes other than P-stress.

Gene	Accession	Class	Description	Correlation	Fold-change (FC)					
					Line 8-27			Line 8-42		
Transcription factors				PSR1	d2	d3	d6	d2	d3	d6
SPL9	Cre16.g683953	I (ABCD)	Squamosa promoter binding	0.89*	**11.8**	**26.3**	**25.2**	1.6	1.8	**6.1**
Bzip1	Cre16.g671200	I (ABCD)	Basic-leucine zipper (bZIP)	**0.96**	**5.5**	**13.8**	**4.9**	1.3	1.9	**2.8**
ZAF1||	Cre26.g756897	III (BD)	PHD-type C-terminal zinc finger	0.29	0.9	1.3	0.8	**2.7**	**4.2**	**10.9**
NOR1	Cre07.g331401	II (ACD)	Orphan nuclear receptor	**0.85**	**2.5**	**9.2**	**5.2**	1.1	1.3	1.4
CSB57	Cre14.g618820	IV (AB)	Transposase DNA-binding	**0.69**	**5.3**	**7.3**	**5.9**	**5.0**	**5.5**	**7.0**
NOR2	Cre15.g637552	IV (AB)	Orphan nuclear receptor	0.20	**4.5**	1.8	0.9	**2.6**	1.5	1.0
RHC1	Cre02.g144802	II (A)	RNA helicase	**0.67**	1.8	**4.2**	2.2	1.0	1.1	1.1
ZF3§	Cre16.g681100	II (ACD)	Zinc/RING finger C3HC4 domain	**0.92**	**2.4**	**4.1**	**2.5**	1.2	1.3	1.5
ZF4	Cre19.g751047	IV (ABD)	CCHC-type zinc finger	-0.04	**3.3**	1.6	2.2	**2.8**	1.7	1.9
ZF1	Cre17.g742800	IV (AB)	CCHC-type C-terminal zinc finger	-0.43	**0.19**	**0.17**	**0.09**	**0.21**	**0.21**	**0.13**
Signal transduction										
MKK5	Cre10.g463500	II (ACD)	Mitogen-activated PKK	**0.79**	**4.9**	**26.0**	**3.9**	1.2	0.9	0.6
ANK1	Cre01.g019550	II (ACD)	Ankryin repeat protein	**0.88**	**5.3**	**14.1**	**6.1**	1.5	1.3	1.3
MRK1	Cre16.g674900	I (ABCD)	Ser/thr protein kinase	**0.84**	**5.3**	**13.0**	**11.9**	1.2	1.4	**3.8**
AUR1	Cre16.g674065	II (ACD)	Aurora protein kinase	**0.75**	**6.6**	**7.1**	**5.2**	1.2	1.1	0.7
MEKK	Cre08.g368600	I (AB)	MEKK-related protein kinase	**0.90**	**6.0**	**6.4**	**5.7**	**2.2**	2.4	1.8
CDPK	Cre02.g114750	III (BCD)	Ca/calmodulin-dependent PK	0.19	1.3	1.4	0.8	**3.3**	**3.7**	**4.1**
XBAT31†	Cre09.g403500	I (ABCD)	E3 ubiquitin ligase	**-0.87**	0.6	**0.14**	**0.06**	0.9	0.8	**0.28**
PK7	Cre08.g369667	IV (AB)	Thr-specific protein kinase	-0.42	0.6	**0.28**	**0.34**	0.6	**0.28**	**0.35**
LAR1	Cre12.g541550	I (ABCD)	Las17-binding actin regulator	0.61	**14.3**	**38.4**	**8.8**	**2.9**	2.1	1.9
BAR	Cre06.g299500	II (A)	Endocytosis regulation	-0.43	**0.24**	**0.15**	0.44	1.6	1.4	1.4
S-stress										
SUTA†	Cre02.g095151	III (BD)	Sulfate-transporting ABC-2 type	0.47	0.7	1.1	1.4	0.7	0.9	**2.6**
SIR†	Cre16.g693202	III (BD)	Sulfite reductase (ferredoxin)	0.45	0.50	1.3	1.4	0.8	1.5	**2.5**
ATS1†	Cre03.g203850	II (ACD)	ATP-sulfurylase	0.27	0.8	0.5	**0.40**	0.6	0.7	1.7
N-stress										
GAT1†	Cre01.g004900	IV (ABCD)	Glutamine amidotransferase	**0.69**	**5.2**	**3.7**	2.2	**4.4**	**2.5**	1.8
NSI1	Cre11.g476026	II (A)	N starvation induced COV1	**0.80**	1.3	**3.0**	**3.3**	1.0	1.1	1.3
Other stress responses										
MGS1	Cre07.g331700	II (ACD)	Minus gamete specific, secretory	**0.69**	**3.0**	**6.6**	2.0	1.4	1.1	0.7
UVE1	Cre12.g505100	III (B)	UV-damage endonuclease	0.56	0.9	1.5	1.4	1.1	0.8	**2.7**
NOS1	Cre16.g683550	II (AC)	NO synthase ferrodoxin reductase	0.44	0.7	1.7	**2.5**	0.7	1.6	1.7
FEA1||	Cre12.g546550	II (AD)	Periplasmic Fe-binding	0.08	**2.2**	1.4	1.3	1.9	1.5	1.6
LCI12	Cre04.g217962	III (B)	Zinc finger, CCHC-type	0.52	1.4	0.8	1.4	**2.2**	1.7	2.6
DNA2	Cre12.g528200	IV (AB)	ATP-dependent helicase (DNA)	-0.44	0.6	0.7	**0.24**	0.5	0.6	**0.32**
METE†	Cre03.g180750	III (BCD)	Vit B12-independent met synthase	0.43	0.54	1.2	1.5	1.8	1.2	**2.1**
PBP1	Cre08.g362600	IV (AB)	Beta-lactamase	-0.33	**0.49**	0.7	**0.32**	**0.38**	0.5	**0.16**
CRD1	Cre12.g537250	II (ACD)	Cyclase in Tetrapyrrole pathway	0.29	0.7	1.8	**0.31**	1.5	1.3	1.8
THB2	Cre14.g615350	I (ABD)	Thylakoid truncated hemoglobin	-0.31	0.8	0.6	**0.28**	1.2	0.7	0.5
GST8‡	Cre11.g467690	II (A)	Glutathione S-transferase	-0.26	**0.08**	**0.04**	**0.04**	0.8	1.5	0.9
HMG1	Cre06.g299550	II (AB)	HMG-CoA reductase	-0.59	**0.09**	**0.04**	**0.15**	1.7	1.4	**2.5**
LTI1	Cre08.g368650	IV (AB)	Lysin-induced mt- secretory	-0.55	**0.03**	**0.02**	**0.05**	**0.03**	**0.04**	**0.04**

*Significant values in bold. Pearson’s correlation coefficients (RPKM, p<0.05, n=3). FC values in relation to UVM4 control (p-adj<0.05, n=3). In the *psr1-1* mutant P-STRESS experiment: Ectopic up†/down‡; Independent up§/down|| (see **Supplementary Data 1**). Gene class is defined as I-IV where Venn class is indicated in parentheses.

In the PCA (**Figure 4**), these genes clustered with, or directly opposed PSR1, indicating a strong positive or negative correlation and hence dependency on PSR1 expression levels (FC data). Class I genes, along with PSR1 associated with PC1, which explained 70% of the variation, suggesting a strong influence. This was further supported by the significant Pearson’s correlation coefficients for most of the Class I genes to PSR1 (RPKM data) (**Tables 2**, **3**). Most Class I genes were induced by PSR1-OE, with some exceptions like XBAT31 and THB2 showing late repression.

Examination of the FC data of the Class I genes indicated that many were upregulated early in both transgenic lines along with PSR1. Particularly those involved in PO_4_^3-^ transport (e.g., PTB2-4, PTA1) and MEKK (**Tables 2**, **3**). This early response occurred even though external PO_4_^3-^ levels (PE) were high at this point in all lines (**Figure 2B**).

Class II genes (Venn AC/D) were only altered in the strongest PSR1-OE line (8-27) and comprised the two largest Venn subsets: set AC/D (also present in the P-STRESS dataset) and set A (specific to OE-248) (**Figure 3C** **)**. Regulation and nutrition were major functional groups as noted in Class I, but more diverse gene functions were present. Although P-stress/assimilation genes were a substantial category among the induced genes in this class, most were P-salvage or P-scavenging (e.g., PHO5/X, SQD3 and GPD2) (**Table 2**; **Figure 4A**). Relatively few potential PO_4_^3-^ transporters were present (i.e., PSTS, PTB12) but significantly, there were four genes linked to PolyP synthesis (**Table 2**).

Examination of the FC data of the Class II genes indicated that virtually all showed a peak in FC data on d3 (**Tables 2**, **3**), at which point external P levels (PE) had been reduced to low levels in line 8-27 (**Figure 2B**). In many cases, the FC peak at d3 was very strong, suggesting dynamic and more complex regulation e.g., for PHO5/PHOX (**Table 2**). Most Class II genes were induced by PSR1 over-expression with only a few exceptions such as the stress-response genes HMG1, GST8 and BAR (regulatory). These showed strong repression, also peaking at d3 (**Table 3**).

**Figure 5:**
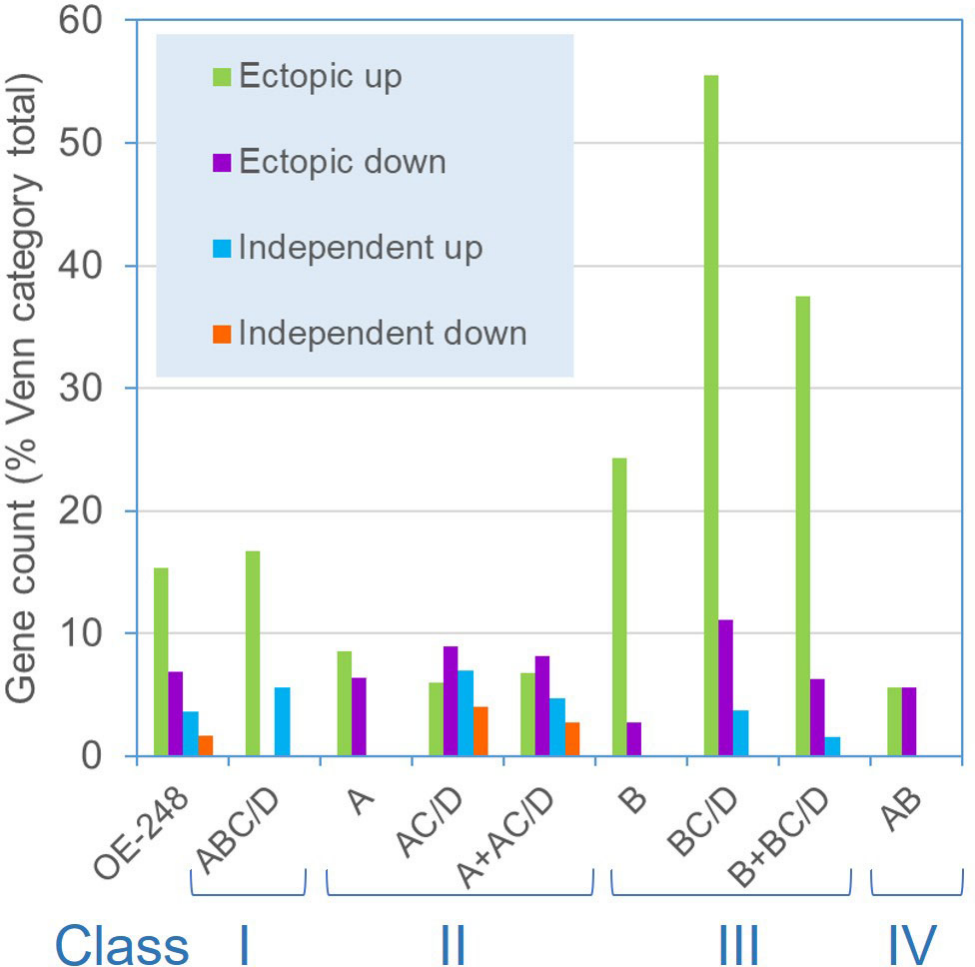
Frequency of PSR1-independent P-stress responses among OE-248 Classes I-IV. Shown are the gene counts for the OE-248 list (and sub-categories) that display P-stress responses in the *psr1-1* mutant within the published P-STRESS dataset (**Supplementary Data 1**)(Bajhaiya et al., 2016). The OE-248 list is subdivided into Venn categories as defined in **Figure 3C**: A (line 8-27) B (line 8-42) C (P-STRESS wild-type “early”) D (P-STRESS wild-type “late”). Class I (ABC/D); Class II (A + AC/D); Class III (B + BC/D) and Class IV (AB). Ectopic genes were those significantly affected (FC>2 or FC<0.5) in the *psr1-1* P-STRESS dataset and either non-significant in wild-type P-STRESS (Venn A, B, AB) or significantly altered from it (ΔFC >2) (Venn XC/D, X=A or B). PSR1-independent genes (Venn XC/D) were (i) similar (ΔFC <2-fold) in wild-type and mutant (ii) in the same direction (up or down) and (iii) biologically significant in both cases (FC>2 or <0.5) in the P-STRESS dataset.

Class III (Venn B or BC/D) genes comprised similar numbers of significantly induced or repressed genes associated only with line 8-42 (sets B or BC/D). This included ZAF1, the strongest “up” gene in line 8-42, albeit late-expressed (**Figure 4A**; **Table 3**). ZAF1 belonged to set BC/D and was therefore validated by its appearance in the P-STRESS dataset (although it was repressed here, **Supplementary Data 1**). ERM1,7 Ca-dependent PO_4_^3-^ transporter channels were significantly repressed in line 8-42 only (**Table 2**; **Figure 4A**). More functionally diverse, this class included two induced S-assimilation genes (SIR and SUTA) (**Table 3**). Class III genes included a high proportion of genes that were ectopically upregulated with P-stress in the absence of PSR1 in the P-STRESS data set (38% cf. 15% for the full OE-248 set) (**Figure 5**).

Class IV genes (Venn set AB) comprised a small number of genes that were affected in both PSR1-OE lines but absent from the published P-STRESS set (and therefore novel) (**Figure 3C**). They consisted entirely of regulatory pathway genes and unknowns, with a bias toward early gene repression rather than induction (**Table 3**). In the PCA (**Figure 4**) the Class IV cluster was oriented equidistant from the two bi-plot clusters, showing similar FC expression data in both lines (**Table 3**). This revealed a lack of quantitative dependence on PSR1 levels for these genes, unlike Class I (ABC/D) which show a closer alignment with line 8-27 and PSR1 (**Figure 4A**).

### 3.8 Most non-P-related stress genes were expressed later than P-related ones

Further multivariate analyses concentrating on actual mRNA levels (RPKM, rather than gene expression changes by FC) revealed that genes for P-stress mitigation consisted of early timing responses (particularly in line 8-27) with distinct regulatory patterns for P-transport, P-sparing and P-salvage. Conversely, those associated with other stresses were late responses in all lines.

**Figure 6:**
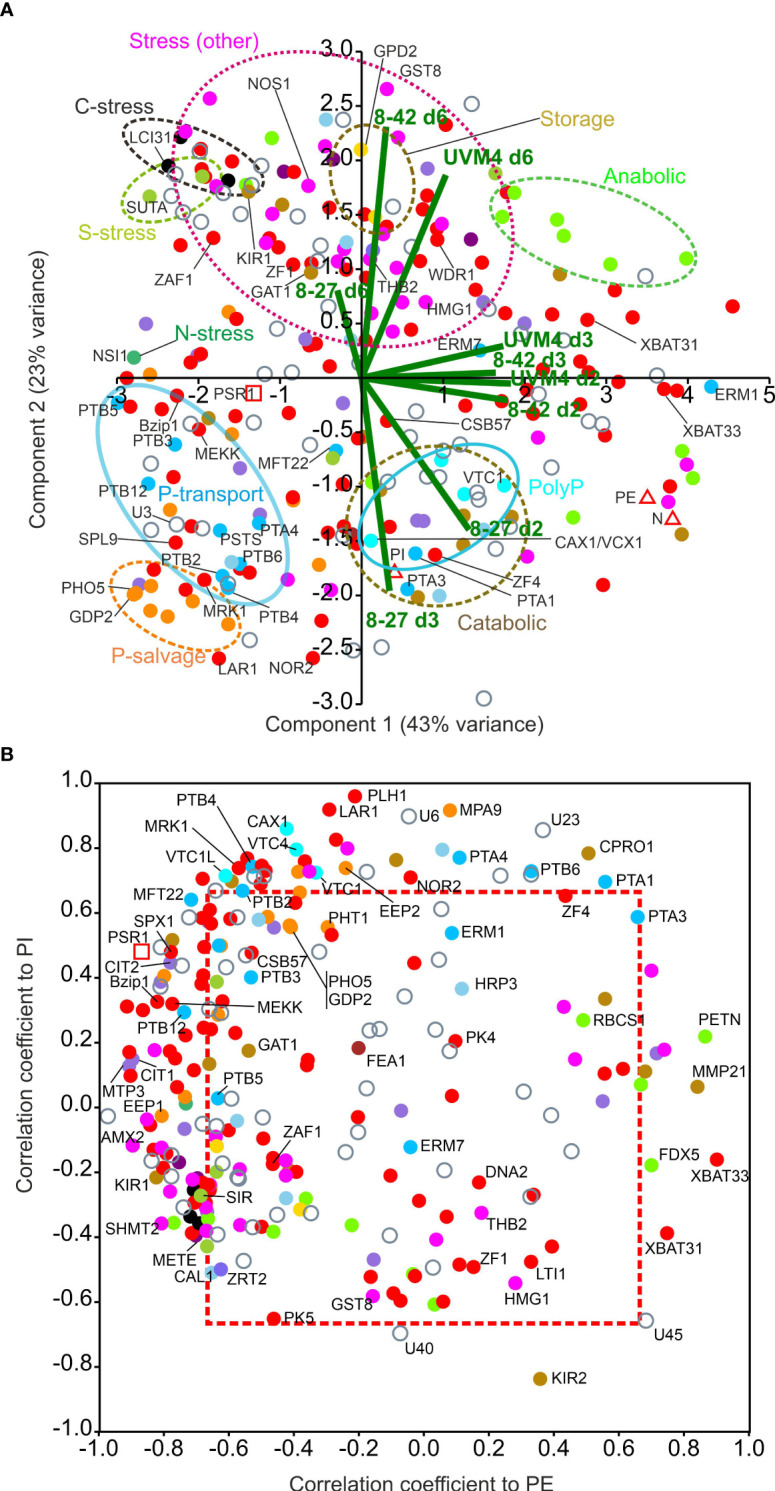
Multivariate analysis of PSR1-OE mRNA levels for OE-248 genes. Patterns of gene expression focusing on temporal mRNA level changes within the OE-248 set. Data points are color-coded for the 17 gene functional processes shown (inset). **(A)** PCA analysis is shown for normalized mean (n=3) RPKM data along with the PE, PI and N measurements (Δ). The biplots for the nine data sets (UVM4 control and PSR1-OE lines 8-27 and 8-42: time points d2, 3, 6; i.e., 9 data points per gene normalized to highest value) are shown (–). Clusters highlighting one specific gene functional process are encircled. **(B)** Plots of Pearson’s correlation coefficients for RPKM data v. PI and v. PE for each OE-248 gene. Coefficients outside the boxed region (---) were significant for PI and/or PE (P<0.05). In all cases mean data was from n=3 culture replicates.

To focus on temporal changes in actual mRNA levels, normalized RPKM data (**Supplementary Data 1**) was used to achieve data separation by PCA in **Figure 6A**. This also allowed co-analysis with physiological measurements (PI, (internal PO_4_^3-^), PE (external PO_4_^3-^) and N (medium N)) and clustered the genes into discrete functional categories (**Figure 6A**). Virtually all the genes for P-stress (early induction) were separated from those for all the other stress responses (N, S, C, Fe, Zn, Cofactor, motility, DNA-repair etc.) and importantly, these were mostly late induced (d6: all lines) (**Figure 6A**; **Supplementary Figure 11A**). The latter group amounted to mostly weaker changes (signaled by their closer clustering to the origin in the earlier PCA in **Figure 4A**) but with some exceptions (e.g., HMG1 and GST8), which underwent larger changes. Situated along the left of the PC1 axis, PSR1 associated P-transport genes, separate from P-salvage genes. By contrast, PolyP synthesis genes associated with internal PO_4_^3-^ levels (PI). Repressed genes in opposition to PSR1 (e.g., XBAT31, 3) associated with PE or N (on the right) (**Figure 6A**).

In **Figure 6B**, correlation coefficients for the OE-248 RPKM data against P-levels: PE and PI, were plotted. A strong bias was seen toward genes that clustered with PSR1. PSR1 itself showed a negative correlation with PE in this figure (p<0.05, **Supplementary Data 1**) and a weak positive PI correlation (NS, **Supplementary Data 1**). These genes were primarily linked to line 8-27 (Venn subsets ABC/D, AC/D or A) i.e., Class I and II (**Supplementary Figure 11B**). A few genes showed regulation in the opposite direction (e.g., FDX5, XBAT31, 3; also, Class I and II) which were repressed by PSR1 and correlated positively with PE (**Figure 6B**; **Supplementary Figure 11B**). A significant positive correlation was seen with PI for the four putative PolyP synthesis genes along with PHL1, a nucleotide triphosphate hydrolase and regulatory genes such as LAR1 (**Figure 6B**; **Supplementary Data 1**). Many (13) Class IV genes (Venn AB) correlated with PI only (e.g., transcription factor NOR2 P<0.05). Some (5) correlated with PSR1 and/or PE, particularly protein kinase MEKK, which was reassigned to Class I (**Figure 6B**; **Supplementary Figure 11B**). Graphs of RPKM v. time are shown for the above genes in **Supplementary Figure 12**.

## 4 Discussion

### 4.1 PSR1-overexpression increased PolyP storage

The ability to switch on luxury-P uptake would greatly facilitate microalgae-based wastewater treatment (Slocombe et al., 2020). Achieving hyperaccumulation of P even when the element is plentiful would avoid the need for stress preconditioning. We examined whether this could be achieved by increasing the levels of a single regulatory gene and if so, what was the impact in terms of gene regulation.

To investigate, we overexpressed PSR1, a global regulator of the P-stress response, generating a spectrum of PSR1 over-expression levels in different transgenic lines. Anticipating that the normal phosphate starvation response (PSR) would be suppressed in the presence of high phosphate levels, PSR1-overexpression was studied under nutrient replete conditions. We established that the P-stress response was weak in the control under these circumstances, both in terms of physiological response and gene expression.

All over-expressor lines tested were successful in completely removing P from the medium by stationary phase, unlike the control. In the strongest line, complete removal of P was achieved by late-log phase, and this was associated by 3-4-fold increases in P-uptake rates and peak biomass P levels (to 8% dw P). Hyperaccumulation was also accompanied by 3-fold increases in PolyP granule diameter. With biomass P at a peak and no P left in the medium, further growth was accompanied by a remobilization of most of the PolyP reserves. This most likely sustained further cell divisions to reach stationary phase (*10*), given that we found no growth penalty associated with PSR1 overexpression.

### 4.2 Mg^2+^ as a dynamic counterion for PolyP accumulation

Close association of uptake rates of Mg^2+^ and P suggested that this cation, rather than Ca^2+^, was acting as a principal counter-ion in PolyP storage. Both are the predominant cations associated with PolyP in *C. reinhardtii* with one report giving higher levels of Mg^2+^ (Ruiz et al., 2001) and in another, they were equal (Komine et al., 2000). Our observation of the 2:1 molar ratio of PO_4_^3-^: Mg^2+^ uptake rates matches the known polymeric stoichiometry of PolyP (Brown and Kornberg, 2004), also suggesting that Mg^2+^ was the main counterion. The enlarged PolyP granules had a diffuse appearance, which might be attributable to a shortage of Mg^2+^. Levels were not actually depleted beyond a low baseline of 3 mg/L from the medium, unlike PO_4_^3-^ however.

### 4.3 PSR1-overexpression mimicked the P-stress response

**Figure 7:**
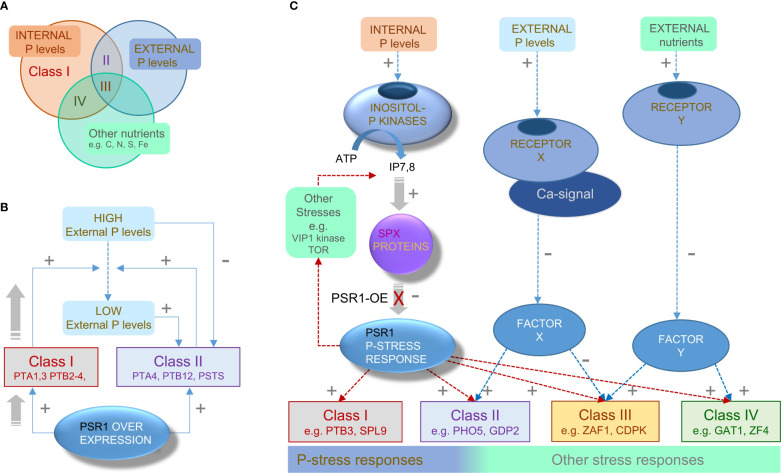
Proposed regulatory networks for PSR1-dependent and PSR1-independent genes. **(A)** Venn diagram illustrating the three nutrient factors or stresses that appear to influence the four Class I-IV subdivisions of the OE-248 set of genes that show significant expression changes (classes defined in **Figures 4A, B**). Each Class I-IV is shown occupying a sector in the Venn diagram indicating the principal nutrient factor or stress that is proposed to influence it. **(B)** Proposed feed-forward mechanism explaining enhanced P-uptake driven by PSR1 over-expression on Class I genes under initially high PE conditions. Activation of Class I P-transporter genes (+) reduces the PE levels which are inhibitory (-) toward Class II genes. Subsequent activation of Class II genes which include P-transporters, further reduces PE. **(C)** Proposed signal network showing left-to-right (i) established PSR1-dependent and (ii) putative PSR1-independent mechanism for P-stress transduction along with (iii) non-P-related stress transduction. We suggest Class I and II genes are primarily PSR1-dependent, but Class II also requires agreement with a PSR1-independent mechanism for perceiving PE. We suggest that Class III genes receive conflicting signals from these two pathways and input from other stress transduction mechanisms. Class IV genes appear to be PSR1-dependent but primarily regulated by non-P-related stress pathways, due to a low correlation of gene expression with PSR1, PE or PI.

Results from a spectrum of PSR1 over-expression levels in different transgenics indicated that P-uptake rates were dependent on the degree of expression. For instance, PSR1 gene mRNA levels were found to have a strong negative correlation with external PO_4_^3-^ levels (PE) and were in proportion to P-uptake rates in PSR1-OE lines. Transgenic over-expression of PSR1 also increased the endogenous wild type PSR1 gene mRNA levels and mimicked the induction of a cohort of genes associated with reported P-stress (Moseley and Grossman, 2009; Bajhaiya et al., 2016) and P-resupply (Plouviez et al., 2021). Altogether 248 genes were found to be significantly altered in gene expression relative to the control (UVM4). Of these, 60% (146) were also seen in one univariate P-stress experiment (Bajhaiya et al., 2016) (but not necessarily described) and the remaining 40% (102) were novel. Despite these similarities, there were major differences in the way four different sub-classes of these genes were affected by PSR1 overexpression. These differences were associated with specific gene functional processes implying that different environmental cues (internal and external PO_4_^3-^ levels and other nutrient stresses) were modifying the gene responses to varying extents (**Figure 7A**).

### 4.4 Proposed feed-forward model accounts for rapid P-uptake in PSR1-OE lines

Evidence suggested that a relatively small group of genes (Class I) were driving the expression of a larger set of genes (Class II) by a feed-forward loop mechanism (**Figure 7B**). This was inferred from multivariate analyses and the timing of gene expression changes. Several Class I genes, mostly consisting of P-transporters (PTA1, 3, 4; PTB2-4) and some putative regulatory genes (e.g., LAR1, MEKK) showed early increases in gene expression (relative to control) in tandem with PSR1 in more than one transgenic line and in the presence of high external PO_4_^3-^ levels (PE). This suggested a lack of PE suppression of gene expression in the case of Class I.

The timing of putative PO_4_^3-^ transporter genes supported the model, whereby low PO_4_^3-^affinity transporter genes were progressively induced and supplanted by those with higher affinity or active transport in PSR1-OE lines: 1^st^ PTA1,3,4 (d2 peak, low affinity; Class I), 2^nd^ PTB2-4 (d2-d3 peak, high affinity; Class I) and 3^rd^ PSTS (active transport: a putative subunit of a prokaryotic type ATP BINDING CASSETTE (ABC) transporter) and PTB12 (high affinity) (both d3 peak; Class II) (**Table 2**, **Supplementary Data 1**) (Shimogawara et al., 1999). Of the PTB genes, only Class I PTB2-4 (cf. PTB5, 6) showed abundant transcript levels (RPKM) as well as high FC’s and were presumably the key players (**Figure 4**; **Supplementary Data 1**). The induction of enzyme activity levels for PTB (higher P-affinity) and suppression of PTA (low affinity) has been noted with P-stress (Shimogawara et al., 1999; Wykoff et al., 1999a).

The model proposes that most of the P-stress related genes (Class II) were inhibited by PE, until levels had been sufficiently reduced by the action of the small group of Class I genes (i.e., the PTA1,3,4 and PTB2-4 P-transporters) (**Figure 7B**). This is supported by the behavior of the PHO genes which encode P-scavenging exophosphatases repressed by PO_4_^3-^ (Moseley et al., 2006). In our multivariate analyses (**Figures 4A, B**) these co-clustered with a large group of PSR1-dependent genes responsible for much of the P-stress response characterized as Class II. These genes tended to peak in expression after the P-biomass transient, for instance one of the strongest relative gene expression (FC) changes was PHO5/X (Class II) which peaked at d3 whereas PTB3,4 peak at d2 (Class I) (**Table 2**). PHO5/X is also one of the most strongly altered genes in P-stress experiments (Moseley et al., 2006; Bajhaiya et al., 2016). The feed-forward aspect of the model is also supported by the induction of Class II putative PO_4_^3-^ transporters (PSTS, PTB12) which could reinforce reductions in PE driven by Class I genes. This could provide an explanation for the rapid P-uptake seen in the transgenic lines, along with complete removal of PO_4_^3-^.

### 4.5 Feed-forward model also accounts for P-overplus

Our model also provides a plausible explanation for the P-overplus phenomenon where P-stress pre-conditioning raises PSR1 levels which then leads to luxury uptake once PO_4_^3-^ is resupplied. Our model suggests that Class I transporter genes (PTB2-4, PTA1,3) would not be suppressed by the resupplied PO_4_^3-^, permitting luxury uptake. This is supported by a recent P-stress/P-resupply experiment where PSR1 and key Class II genes (e.g., PHOX/5, PTB12, MPA2) were immediately suppressed by P-resupply after 1 h but key Class I genes (PTB2-4) were not suppressed until 5-24 h or were increased after 1 h (PTA1,3)(Plouviez et al., 2021).

### 4.6 Regulatory model incorporates a PSR1-independent external P sensor

Assembling a gene model of the P-stress sensing apparatus integrated the findings in this work with knowledge in the literature (**Figure 7C**). The model incorporates the well-established Inositol PolyP (InsP7, 8) pathway, which is presumed to operate in *C. reinhardtii* for the detection of internal PO_4_^3-^ (Lorenzo-Orts et al., 2020). We propose that for Class I genes, increasing PSR1 levels overcomes InsP7, 8-mediated inhibition that is signaling an ample P-supply, perhaps by titrating out the SPX1 protein. In vascular plants, the PSR1 homologue PHR1 co-ordinates multiple aspects of the response to low phosphate (Rubio et al., 2001). Under high PO_4_^3-^ it is prevented from binding its target site in the promoter of downstream genes by inositol P dependent binding to SPX1 (Puga et al., 2014; Wild et al., 2016). In the current study the SPX1 gene was itself increased by PSR1 overexpression but this was not an early response and was also relatively weak (**Table 2**).

We propose that PSR1-dependent Class II genes (e.g., PHO5/X) are also regulated by a further PSR1-independent inhibitory mechanism sensing the external PO_4_^3-^ levels (PE) (**Figure 7C**). This would render the genes initially insensitive to PSR1 increases until PE levels were decreased by the action of Class I PTB2-4. This mechanism could also explain the occurrence of a minority of PSR1-independent genes that are reportedly altered in P-stress (Bajhaiya et al., 2016). In fact an external PO_4_^3-^ receptor has been proposed in diatoms on the basis of Ca-mediated signaling for PO_4_^3-^ (Helliwell et al., 2021) culminating in gene expression changes through unknown means (Factor X) (**Figure 7C**). According to our model, PSR1-independent genes (Bajhaiya et al., 2016) would be regulated by the external PO_4_^3-^ sensor pathway only. This could be readily tested by using Ca-signaling inhibitors followed by mRNA profiling of candidate Class I and Class II genes. Additionally, the TOR signaling complex, which integrates nutrient status to control growth and various anabolic or catabolic pathways, has been placed downstream of PSR1 (via LTS8) (Couso et al., 2020). TOR also interacts with the VIP1 kinase which produces InsP7,8 so could potentially act upstream of PSR1 via SPX1 (**Figure 7C**) (Couso et al., 2021).

### 4.7 PSR1 influences non-P-related stress and general stress response genes

The global nature of PSR1 beyond P-homeostasis has been indicated (Ngan et al., 2015) as well as a newfound role for PolyP in mitigating S-stress through utilization of excess ATP (Sanz-Luque et al., 2020). We found that PSR1 over-expression also led to changes in genes that appear to alleviate other nutrient stresses (C, N, S, Zn, Fe and vitamin cofactors) or herald general stress responses in *C. reinhardtii* (e.g., DNA-repair, reproduction, motility, defense, storage products etc.). Most of the actual mRNA levels for these genes increased later in the time course than those of the P-stress genes. This suggested that various stress signals associated with stationary phase might integrate with PSR1-mediated signaling, hence the more complex modes of regulation in Class II, III, IV shown in the model (**Figure 7C**). For instance, nutrient stress genes (C, S, N, Fe etc.) are affected during batch culture of the UVM4 control, whereas P-stress genes were under-represented by comparison. C-stress was a prominent factor and early reductions were also seen in protein translation gene expression.

A good example of this complex regulation was GAT1 which experienced a strong early response to PSR1 over-expression (**Table 3**) in 2 transgenic lines irrespective of PSR1 levels (Class IV). Transcript abundance was greater at later stages for all lines, however. GAT1 encodes a peptidase that might act to alleviate N-stress and a potential drop in key amino acids. This has in turn been linked to C-stress (TOR-kinase mediated) (Mallén-Ponce et al., 2022), which might account for early reduction in the protein translation genes seen in all lines. In addition, GST8 a glutathione S-reductase (oxidative stress) and HMG-CoA reductase (HMG1) were both strongly repressed with PSR1-overexpression at mid-culture. These were postulated to be PE-dependent (Class II). Reduction of sterol synthesis (i.e. ergosterol) could be a consequence of repressing the reductase, and this is a known stress response in other organisms (Montañés et al., 2011).

Many of the non-P-related stress genes fell into Class III which were paradoxically more highly regulated with weaker PSR1 over-expression. For instance, S-stress: SUTA and SIR; low-C stress: LCI12 and cofactor auxotrophy METE (bypasses a requirement for vitamin B_12_) (Helliwell et al., 2016). The aforementioned S-stress function genes induced by PSR1 over-expression were not among the most strongly induced genes reported for a univariate S-stress experiment (González-Ballester et al., 2010), whereas ATS1 (Class II), which was induced strongly in this report showed significant repression in our work (**Table 3**). Therefore, a somewhat different complement of genes was being influenced by PSR1 over-expression in our work.

Although Class III genes were specific to the weak PSR1-OE line, a significant amount (42%) were published P-stress-affected genes (Bajhaiya et al., 2016) and we found many (38%) to be ectopically regulated in the *psr1-1* mutant (Bajhaiya et al., 2016). This was mostly ectopic induction, where presumably PSR1 opposes induction and a PSR1-independent P-stress mechanism is responsible for the increase in the mutant (**Figure 7C**). Conversely, the most strongly induced Class III gene, ZAF1 (a transcription factor) was repressed in a PSR1-independent fashion by P-stress (Bajhaiya et al., 2016). Therefore, it could instead be induced by PSR1 but repressed by a PSR1-independent P-stress mechanism (**Table 3**). We propose that in Class III there might be a conflict between the two mechanisms of P-stress perception that could act to shift the focus more exclusively to P-homeostasis genes when P-stress dominates (**Figure 7C**). For instance, when PSR1 is over-expressed at higher levels we suggest Class III genes are dampened down because PE is reduced faster.

Expression levels of the Class IV genes typically lacked correlation to PSR1 or PE, and gene mRNA levels mostly increased in stationary phase suggesting that they might be influenced by other nutrient stresses (represented in **Figure 7C** by unknown Factor Y).

### 4.8 Accounting for the transience of stored PolyP

One factor that could have impacted PolyP stability was the transcriptional control of the synthesis pathway. Several Class II genes were linked with PolyP production, for instance the CDF family cation transporter MTP4 (35-fold) and Chromate ion transporters CIT1, 2 (5-fold) which could be responsible for the rapid Mg^2+^ uptake. Somewhat lower induction levels were noted for a Ca^2+^/H^+^ vacuolar antiporter (CAX1/VTC1) along with three putative PolyP synthesis genes (VTC1, VTC1L and VTC4) (2-3-fold). Interestingly, a triphosphate hydrolase (PLH1) (6-fold) co-clustered with this latter group of genes, where all exhibited a close positive correlation with internal P_i_ (PI) levels.

The transient nature of expression in these and other Class II genes may have contributed to PolyP remobilization. The sharp decrease (particularly evident for genes such as PHO5/X) suggests a negative feedback mechanism and/or sensitivity to decreases in PSR1-YFP protein levels. The latter was supported by use of protein translation inhibitors in relation to PHO5/X (Bajhaiya et al., 2016). It is plausible that PSR1-YFP is removed by post-translational means once PO_4_^3-^ is removed from the medium, for instance. Expression of the transgene construct, which was driven by a *C. reinhardtii* PSAD light-regulated promoter might also have contributed. The wild-type PSAD gene levels dropped by 35-40% over the experiment (**Supplementary Data 1**), albeit without variation between lines. The endogenous PSR1 gene transcript continued to increase in expression, as external PO_4_^3-^ was depleted, in all lines. In the literature however, upregulation of the PSR1 gene mRNA has been shown to be transient in univariate stress experiments (P, N, S) (Wykoff et al., 1999b; Ngan et al., 2015; Bajhaiya et al., 2016).

Other regulatory pathways were found that might account for transient gene expression, such as THB2 (a Class I regulatory gene) which was late-repressed by PSR1 over-expression. This truncated hemoglobin is thought to act as an NO scavenger (Grinko et al., 2021). In addition, we saw late induction of NOS1 (3-fold) a possible flavodoxin/nitric oxide synthase with a ferrodoxin reductase-type FAD-binding domain. Together these changes were consistent with NO production with P-stress (Grinko et al., 2021). Switching off THB2 represses P-stress induction of genes such as PHO5/X, providing a possible negative feedback mechanism that might also drive PolyP remobilization (Filina et al., 2019; Grinko et al., 2021). Poly P synthesis and mobilization are also regulated directly at the enzymatic level by the binding of inositol phosphates (InsP7, 8) to SPX domains attached to VTC proteins and PTC1 (**Figure 7C**) (at least in yeast) (Austin and Mayer, 2020; Wang et al., 2021). In a recent study, combining PSR1-OE with a PTC1 knockout increased P-uptake and storage further (Wang et al., 2023), although remobilization still took place in the absence of external P.

## 5 Conclusions

In this study we demonstrated that over-expressing a single gene (PSR1) led to a remodeling of metabolism that induced luxury P uptake. We found that enhanced P-uptake was accompanied by an accumulation of large PolyP storage granules. Our data strongly implicated Mg^2+^ as the principal counter ion for P-storage in this form. We also identified a feed-forward mechanism where elevated PSR1 promotes induction of a small set of ‘*driver genes*’ at high PO_4_^3-^ levels. These go on to reduce external PO_4_^3-^ levels, allowing the induction of P-repressed genes responsible for further P-uptake. This model also explains the P-overplus phenomenon where luxury P-uptake is induced by P-stress followed by P-resupply. Collectively, these results will accelerate progress toward circular bioeconomy algal solutions for wastewater treatment or eutrophic waterbody bioremediation.

## Data availability statement

The datasets presented in this study can be found in online repositories. The names of the repository/repositories and accession number(s) can be found here: https://doi.org/10.5518/1217.

## Author contributions

Project concept: AB and MC-V. Transgenic constructs and transformation: LC, PM, and MD. Confocal microscopy: TZ-B and LC. Physiology experiments: TZ-B, SS, and LC. Western blotting, ICMS & assays: TZ-B. RNA-seq analyses & models: SS. Investigation: TZ-B, SS, and LC. Supervision: AB, MC-V, and AS. Figures: SS and TZ-B. Writing—original draft: SS and TZ-B. Writing—review & editing: SS, AB, MD, AS, PM, TZ-B, MC-V, and LC. All authors contributed to the article and approved the submitted version.
