# Supplementary material for: Overexpression of PSR1 in Chlamydomonas reinhardtii induces luxury phosphorus uptake
Source: Front Plant Sci. 2023 Jul 27;14:1208168. doi: 10.3389/fpls.2023.1208168 (PMC10413257; doi:10.3389/fpls.2023.1208168)
Supplement: Supplementary file 1 [file DataSheet_1.pdf]

Supplementary Material

Overexpression of PSR1 in *Chlamydomonas reinhardtii* induces luxury phosphorus uptake.

Stephen P. Slocombe[1], Tatiana Zúñiga-Burgos[1,2], Lili Chu[1], Payam Mehrshahi[3], Matthew P. Davey[3], Alison G. Smith[3], Miller Alonso Camargo-Valero[2,4*], Alison Baker[1*]

*** Correspondence:** Corresponding Author: a.baker@leeds.ac.uk; m.a.camargo-valero@leeds.ac.uk

1 Supplementary Data

Supplementary Data 1 (excel file)

2 Supplementary Figures and Tables

Supplementary Fig. 1-12 and Supplementary Tables 1-3.

2 Supplementary Figures and tables

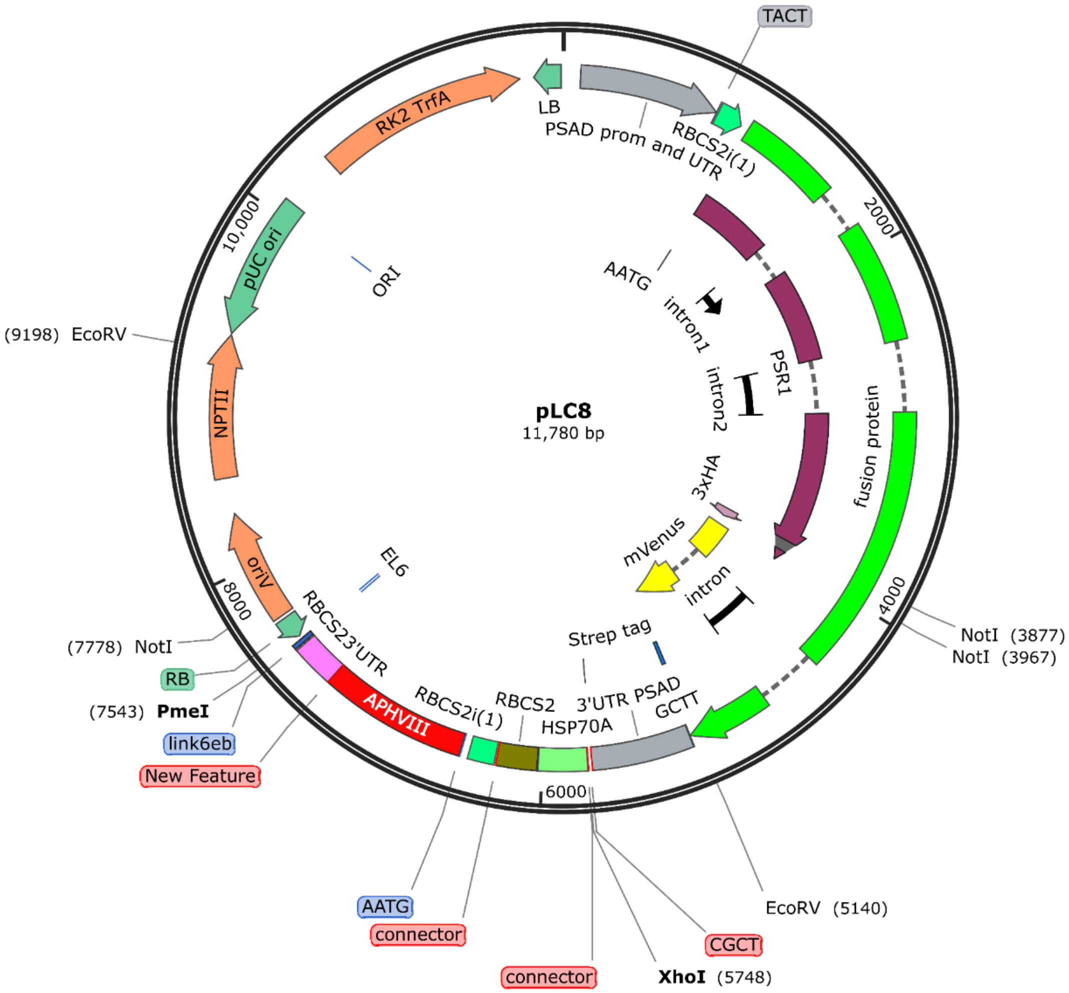

Supplementary Figure 1. Plasmid map of pCL8, the PSR1-OE construct. The pLC8 construct was based on the pUC57 backbone, which provided kanamycin resistance (nptII) for microbial selection. From the top, the PSR1-OE component included the PSAD constitutive promoter with an RBCS2i enhancer driving the PSR1 gene. The latter was terminally fused to a 3X HA tag then YFP (mVenus) followed by 3'UTR of PSAD. Downstream of this gene was a second gene construct for Paromycin resistance for enabling selection of algal transformants.

PSR1(HA)VenusStrepII Fusion protein

1048 amino acids; MW 109363; pI 5.35

MDKAERAAGGPNAASEDDWLLEFWPEPAADFPAPVAPMLSQHQDAAQLPEAMPQQQGLALGGYGLTQ
QPSDFMQTGMPGFDAFSSGKAATLGLPLLADPQRASTDGASALMNAAQQSSEYMLAPGMGGMPHLLA
PSVGTALPGTGHTGFADLSMGGMAGGIPGLGGPGIMHGQYFMQPQRAATGPAKSRLRWTPELHNRFV
NAVNSLGGPDKATPKGILKLMGVDGLTIYHIKSHLQKYRLNIRLPGESGLAGDSADGSDGERSDGEG
GVRRATSLERADTMSGMAGGAAAALGRAGGTPGGALISPGLAGGTSSTGGMAAGGGGGGLVTEPSI
SRGTVLNAAGAVATAAPAAAAPAGGSAAVKRPAGTSLSSGSTASATRRNLEEALLFQMELQKKLHEQ
LETQRQLQLSLEAHGRYIASLMEQEGLTSRLPELSGGAPAAAPVAAGGAAGGMIAPPPPQQQLQHQP
QLLQPQGSLPAGGSSEAHAAAGAGTMVVHQQQQHVHHHQQQQVQMQQHARHCDTCGAGGAGGAPS
GGSSMQQLQAAEQQRTELVVAGRLGSMPAPASSSPLAGQAHQQQPLAGGAAHLVHVHSHTPGGQPHV
QHQDAFAGAATAAAHASPGLPQSHSHLLPADLSSNAGPDTSAGQIKPEPDMSQQQQQQEQQEAEQLA
QGLLNDSSAGAGAVSGSDGGGLGDFDFGDFGDLDGGAQGGLLGPGDLIGIAELEAAAAYPYDVPDYA
YPYDVPDYAYPYDVPDYAAAAHEQQQEQEHDPLDADRAKRQRVEPSSHMRSDVIEGRVSKGEELFTG
VVPILVELDGDVNGHKFSVSGEGEGDATYGKLTLKLICTTGKLPVPWPTLVTTLGYGLQCFARYPDH
MKQHDFFKSAMPEGYVQERTIFFKDDGNYKTRAEVKFEGDTLVNRIELKGIDFKEDGNILGHKLEYN
YNSHNVYITADKQKNGIKANFKIRHNIEDGGVQLADHYQQNTPIGDGPVLLPDNHYLSYQSKLSKDP
NEKRDHMVLLEFVTAAGITLGMDELYKIEGRDIEFWSHPQFEK*

Supplementary Figure 2. Amino acid sequence of PSR1-OE construct fusion protein. The fusion protein was of predicted size 109 kDa and consisted of an N-terminal fusion of PSR1 (MDK…VEP) (*C. reinhardtii* v5.6 Accession: Cre12.g495100.t1.2) containing a 3xHA tag inserted towards the end in a stretch of Alanines (YPY…DYA), with a C-terminal mVenus YFP (MRS…FEK).

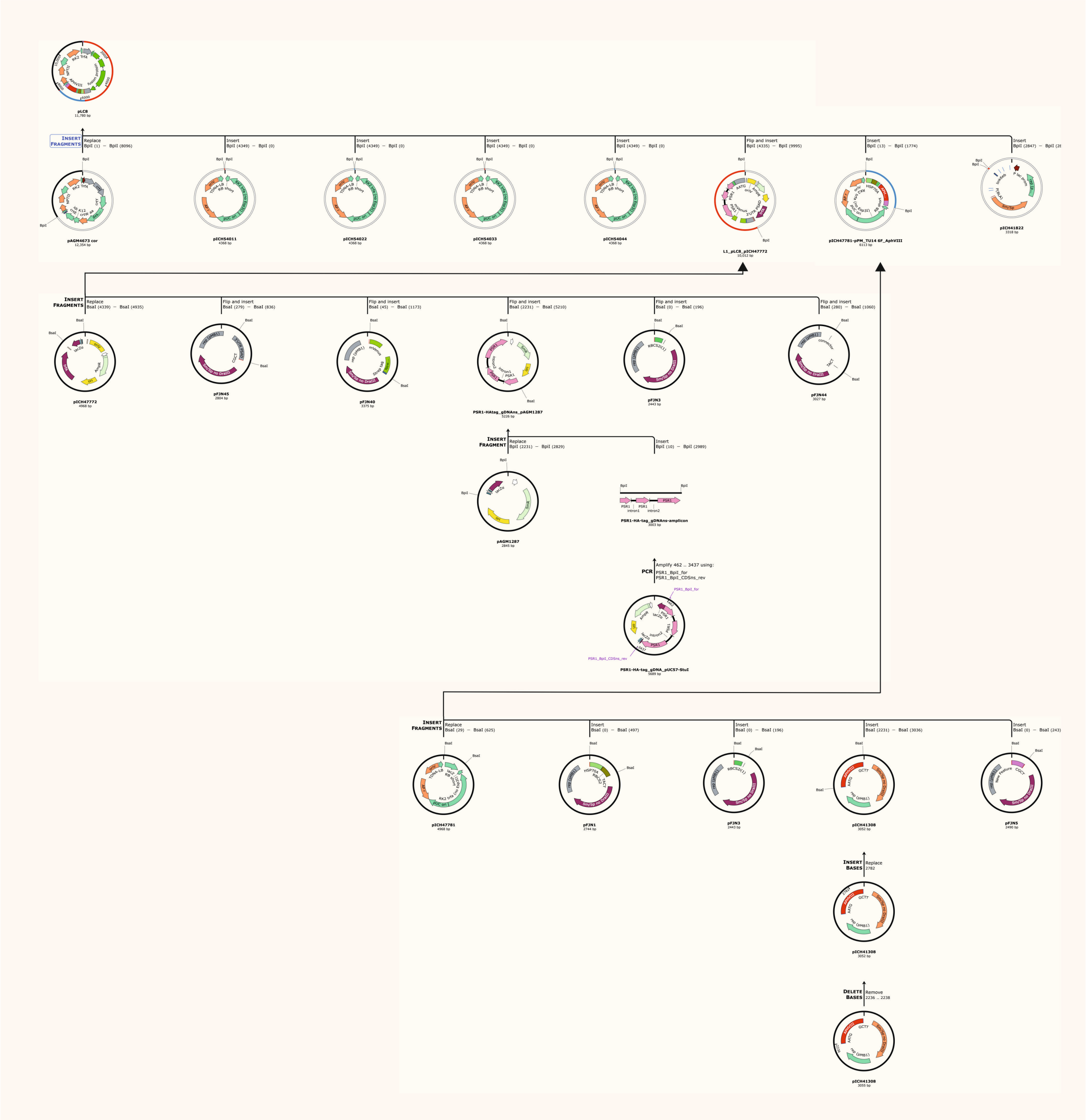

Supplementary Figure 3. Full cloning history of the pCL8 construct (zoom into image). The PSR1 gene (Cre12.g495100.t1.1) with an inserted 3xHA-tag was synthesized and cloned into pUC57 via the StuI restriction site. This plasmid was then used as a template for Golden Gate-based cloning with the full cloning history described in **Materials and Methods.**

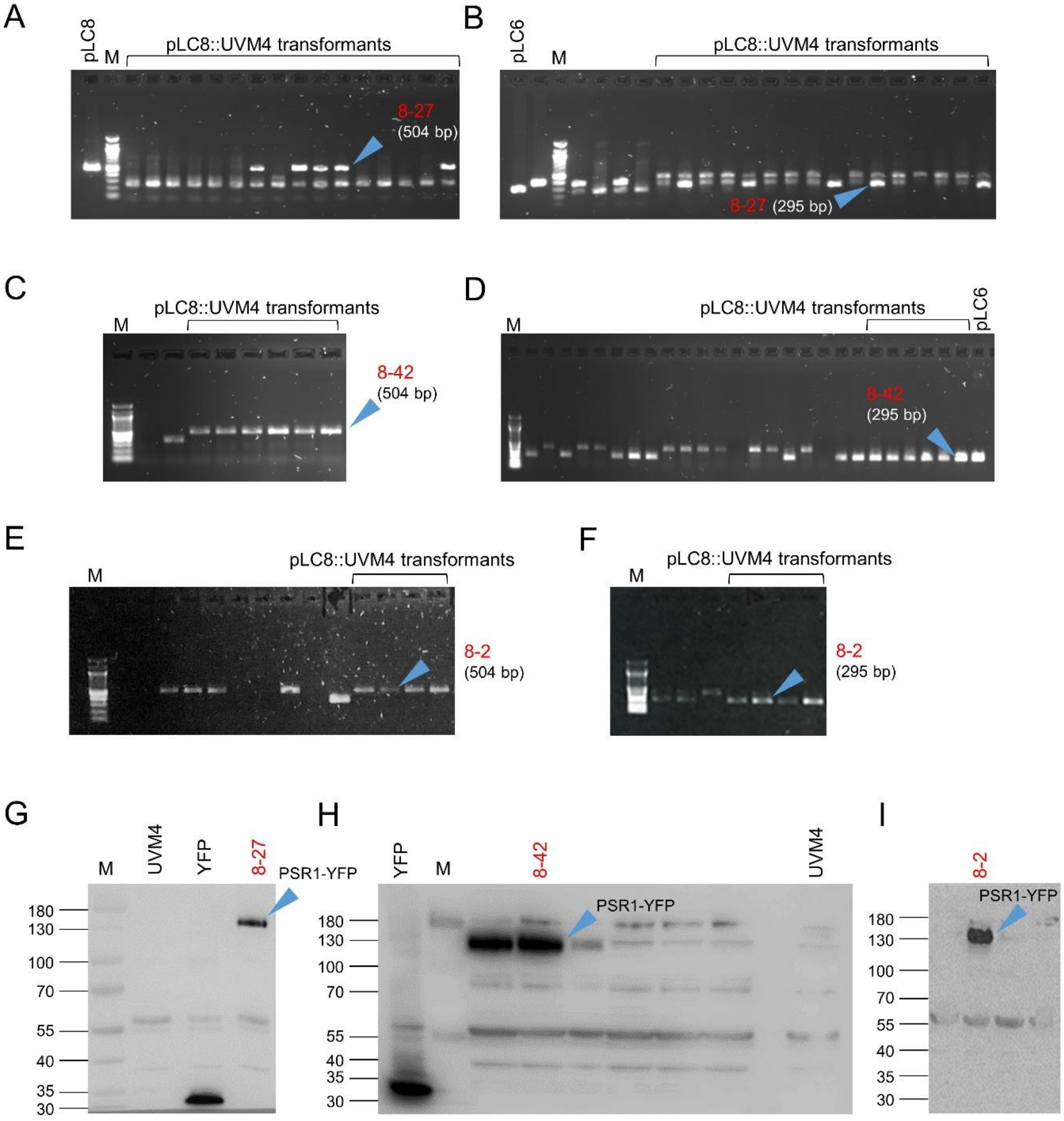

Supplementary Figure 4. Screening for PSR1-YFP construct and fusion protein in transgenic lines. Colonies from three independent transformations of *C. reinhardtii* were screened by (**A-F**) colony PCR for the presence of the PSR1-YFP construct and (**G-I**) Western blotting for presence of the PSR1-YFP fusion protein. (**A, B, G**) **Line 8-27** is highlighted from transformation date 23-11-2017. (**C, D, H**) **Line 8-42** is highlighted from transformation date 22-03-2018. (**E, F, I**) **Line 8-2** is highlighted from transformation date 26-04-2018. (**A, C, E**) The PSR1 portion of the construct was detected by PCR primers LC40 and LC42 giving rise to a 504 bp product, utilizing the pLC8 plasmid DNA as a positive control. (**B, D, F**) The Venus YFP portion of the construct was detected using primers LC43 and LC45 generating a 295 bp product, using pLC6 plasmid DNA as a positive control. (**G-I**) Western blot analysis of protein extracts from the selected colonies for presence of the fusion protein using anti-YFP antibody (predicted size 109.4 kDa). The controls were untransformed UVM4 (negative) and UVM4 transformed with the YFP construct only (positive). Chlorophyll loadings were 2.5 µg (**G, I**) and 5.0 µg (**H**). Marker (M), primers are listed in **Supplementary Table 1**.

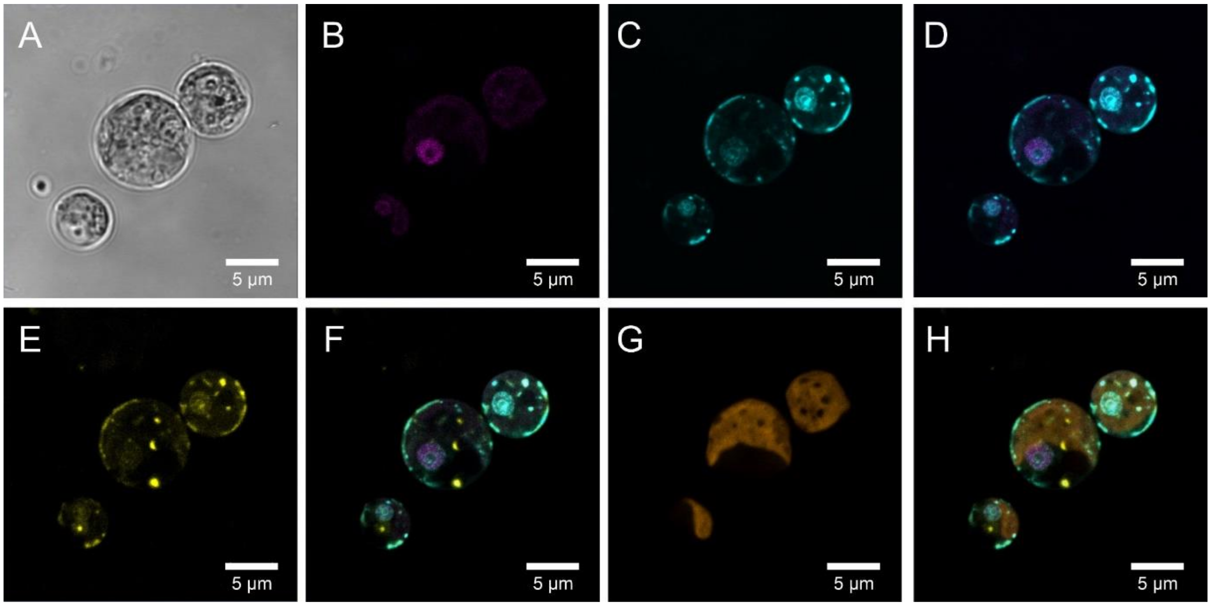

Supplementary Figure 5. **Intracellular targeting of PSR1-YFP fusion protein as determined by fluorescence confocal microscopy**. The intracellular location of the PSR1-YFP fusion protein shown in (**A-H**) for representative cells from PSR1-OE line 8-42 grown in TAP media. Similar data for the expression of the independently transformed PSR1-OE line 8-27 is shown in **Figure 1**. (**A**) bright-field images indicating cell diameter. (**B**) Venus-YFP signal (Emission λ 520-550 nm: magenta) indicating location to the nucleus which was identified by DAPI-DNA fluorescence (Emission λ 420-475 nm: cyan) (**C**) followed by co-localization of the DAPI and YFP signals in the merged image (**D**). The PolyPhosphate (PolyP) granules are indicated by the DAPI-PolyP (Emission λ 535-575nm: yellow) (**E**). These are shown to be separate entities from those identified by DNA-DAPI staining in the merged image (**F**). Chlorophyll UV-fluorescence (Emission λ 670-720 nm: orange) indicating the single large cup-shaped chloroplast (**G**) and the merged image (**H**) which excludes the YFP and PolyP signals from this organelle, placing the PolyP signal to the periphery of the dark central region of the cell (vacuole).

A

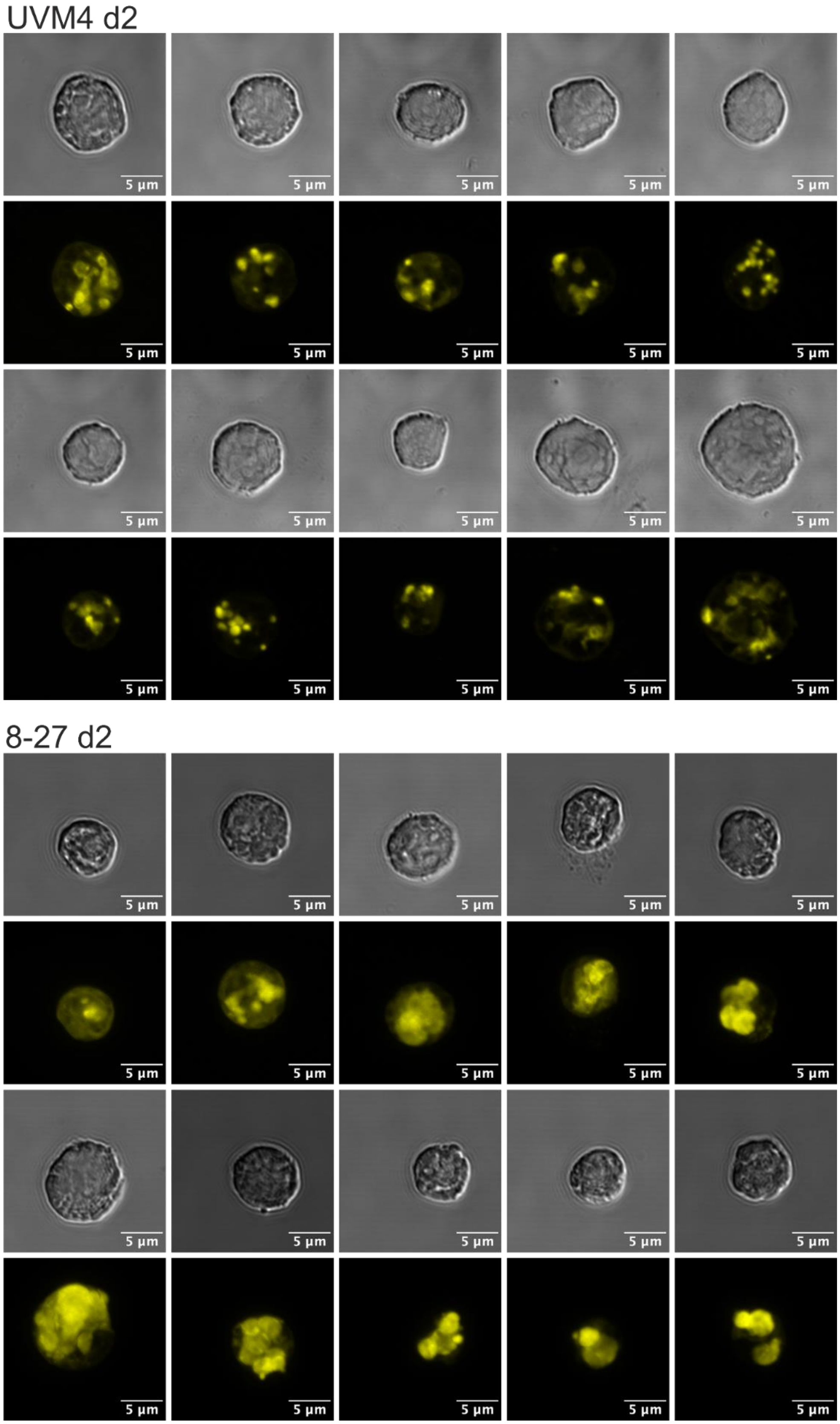

Supplementary Figure 6A. **Increases in PolyP storage granule size as determined by fluorescence confocal microscopy associated with PSR1 over-expression (d2)**. Increases in PolyP storage granule size as determined by fluorescence confocal microscopy associated with PSR1-OE. Displayed are differences in the accumulation of PolyP in cells from a batch culture time course in TAP media for time points d2 where 10 representative cells are compared for control line UVM4 (top) and PSR1-OE line 8-27 (bottom). For each cell the bright field (top) and the DAPI-PolyP signal (bottom) is shown (Emission λ 535-575nm).

B

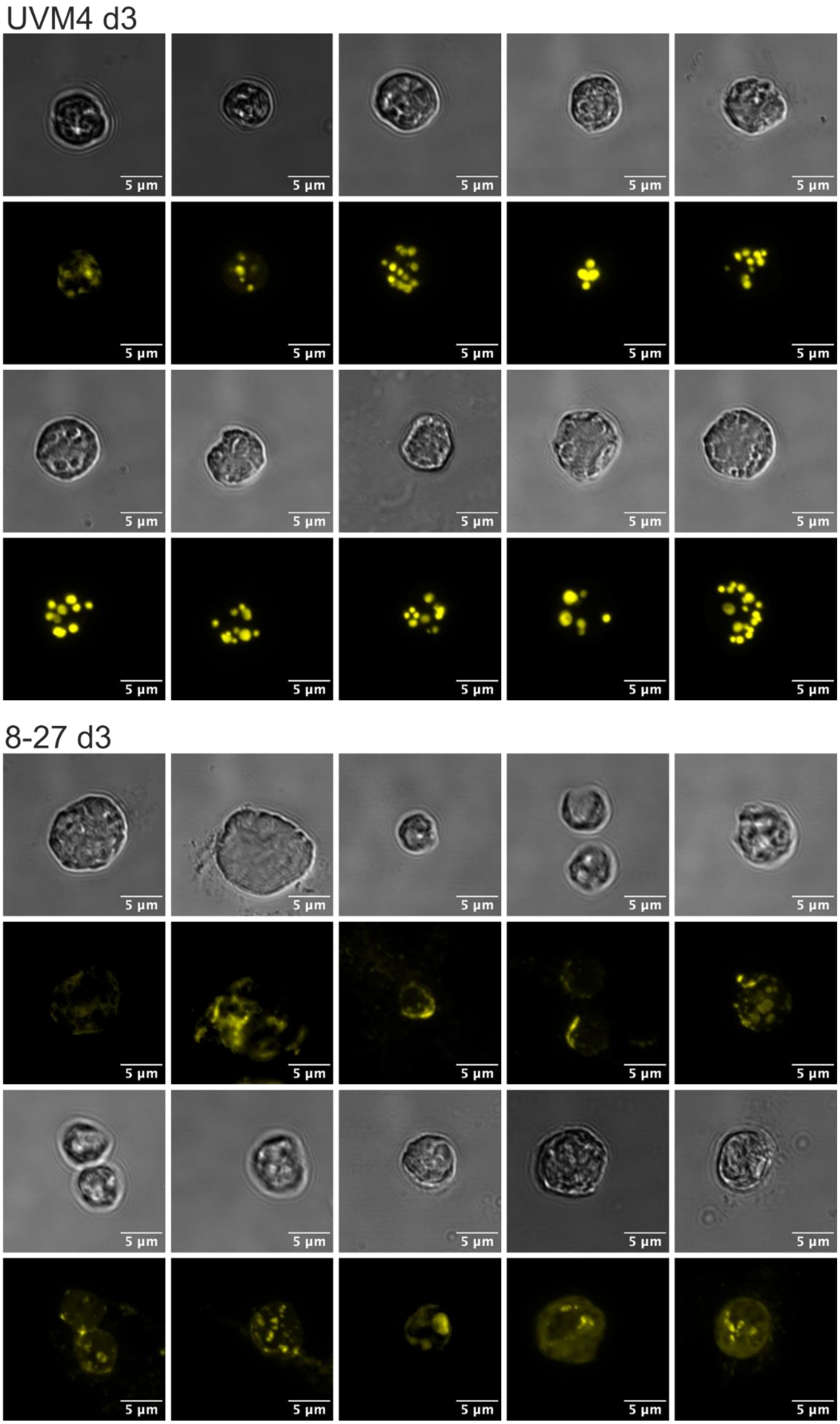

Supplementary Figure 6B. **Increases in PolyP storage granule size as determined by fluorescence confocal microscopy associated with PSR1 over-expression (d3)**. Increases in PolyP storage granule size as determined by fluorescence confocal microscopy associated with PSR1-OE. Displayed are differences in the accumulation of PolyP in cells from a batch culture time course in TAP media for time points d3 where 10 representative cells are compared for control line UVM4 (top) and PSR1-OE line 8-27 (bottom). For each cell the bright field (top) and the DAPI-PolyP signal (bottom) is shown (Emission λ 535-575nm).

C

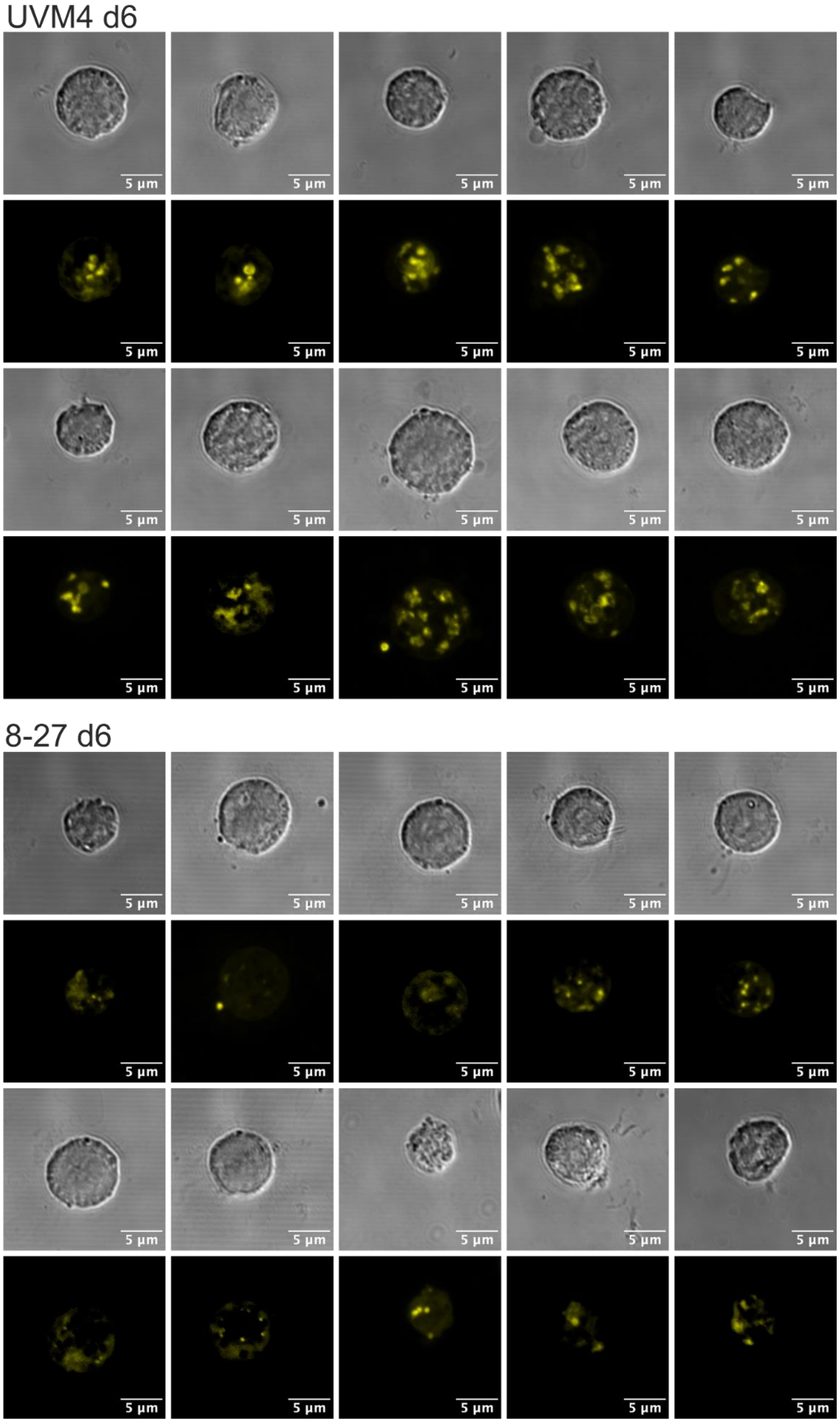

Supplementary Figure 6C. **Increases in PolyP storage granule size as determined by fluorescence confocal microscopy associated with PSR1 over-expression (d6)**. Increases in PolyP storage granule size as determined by fluorescence confocal microscopy associated with PSR1-OE. Displayed are differences in the accumulation of PolyP in cells from a batch culture time course in TAP media for time points d6 where 10 representative cells are compared for control line UVM4 (top) and PSR1-OE line 8-27 (bottom). For each cell the bright field (top) and the DAPI-PolyP signal (bottom) is shown (Emission λ 535-575nm).

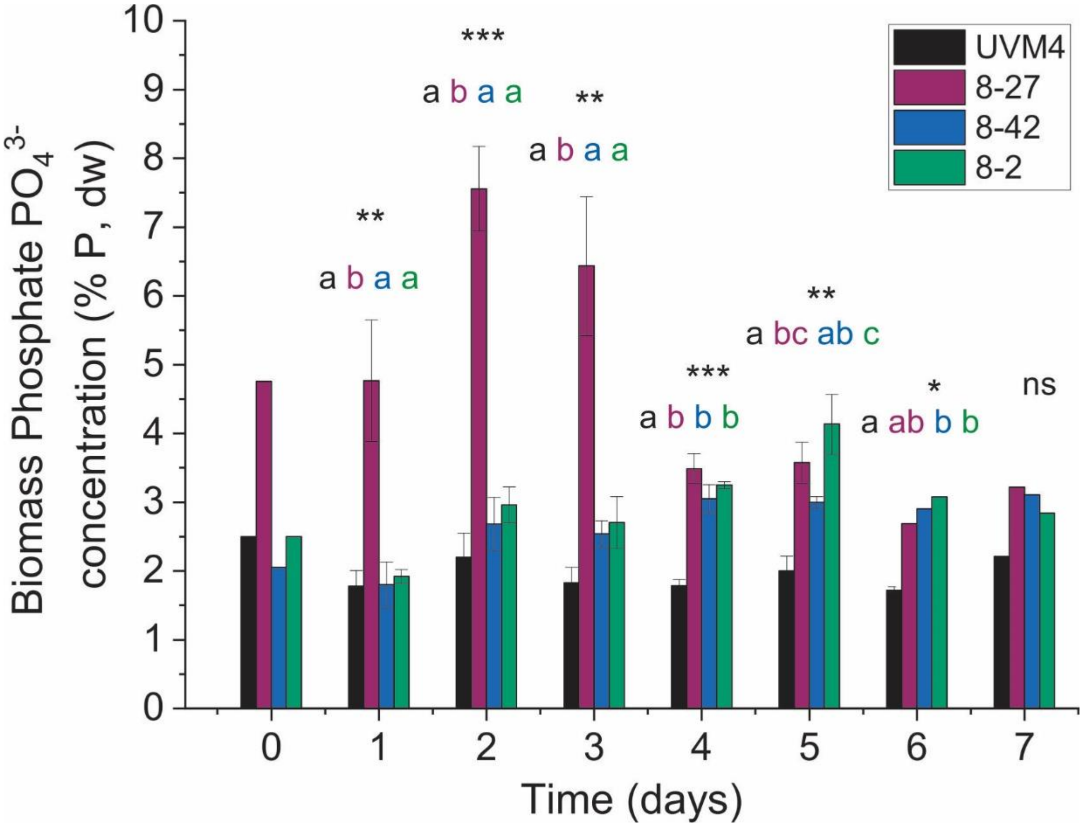

Supplementary Figure 7. **Statistical analysis of enhanced P in biomass accumulation in PSR1-OE lines.** Measurements P in biomass composition at different stages of algal growth. Three transgenic PSR1-OE-lines, along with the untransformed UVM4 background control, were cultivated under small-scale batch culture conditions in TAP media (30 mg/L P ~ 1mM) in continuous light. Statistical differences were analysed by One-way ANOVA to validate the replicates and determine differences between the transgenic lines and the control: ns (not significant), * ($P<0.05$), ** ($P<0.01$), *** ($P<0.001$) and **** ($P<0.0001$). Where $p<0.05$ (One-way ANOVA), multi-comparison statistical analysis was carried out between each of the four lines with a Tukey HSD test (p-value<0.05). Letters from a-d were assigned to group the lines with no significant differences on each day of cultivation.

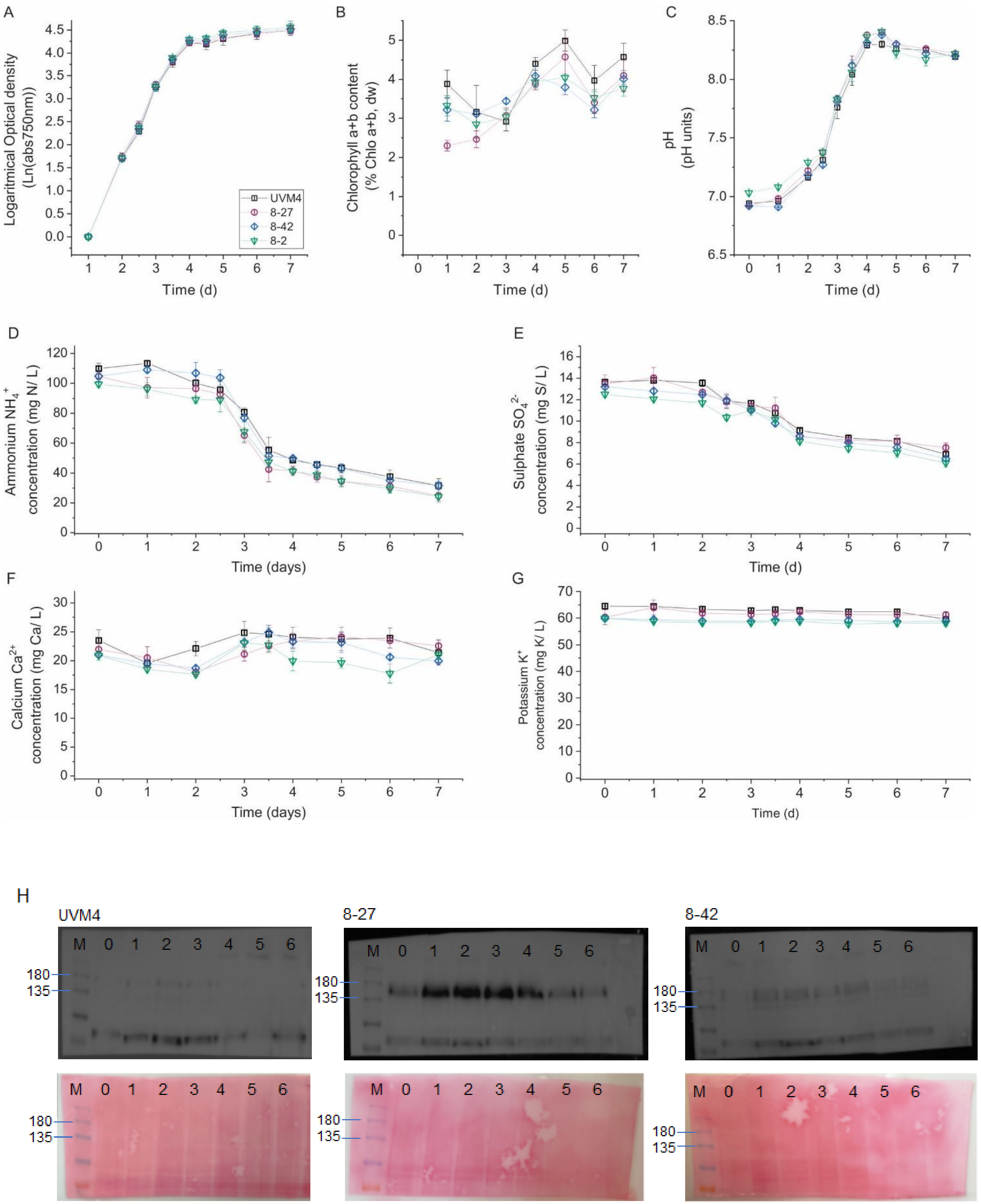

A
Logaritmical Optical density (Ln(abs750nm))
Time (d)
UVM4
8-27
8-42
8-2
B
Chlorophyll a+b content (% Chlo a+b, dw)
Time (d)
C
pH (pH units)
Time (d)
D
Ammonium NH4+ concentration (mg N/ L)
Time (days)
E
Sulphate SO42- concentration (mg S/ L)
Time (d)
F
Calcium Ca2+ concentration (mg Ca/ L)
Time (days)
G
Potassium K+ concentration (mg K/ L)
Time (d)
H
UVM4
8-27
8-42
M 0 1 2 3 4 5 6
180
135

Supplementary Figure 8. Physiological parameters and western blot analysis of transgenic lines and UVM4 control. Three transgenic PSR1-OE-lines, along with untransformed UVM4 background control, were cultivated under small-scale batch culture conditions in TAP media under continuous light. (**A**) Growth rates (log plot of optical density (OD)), (**B**) biomass chl a+b levels, (**C**) pH, (**D**) Medium N levels, (**E**) medium S levels, (**F**) medium Ca levels and (**G**) medium K levels. (**H**) Depiction of complete Western blots from **Figure 2E** showing time-course data (d 0-6) for lines UVM4, 8-27 and 8-42: (top) anti-YFP antibody signal and (bottom) total protein staining with Ponceau Red. Equal chlorophyll loadings (10 εg) were loaded where d0 refers to the starter culture prior to dilution. PSR1-YFP fusion migrated just above the 135 kDa marker (M). Anti-YFP antibody signal indicates that the PSR1-YFP protein was not detected in the control line UVM4, was strongly expressed in line 8-27 (peaking at d2-d3) and was expressed to a lesser extent in line 8-42 (peaking at d1-4). Ponceau Red stains indicate that protein loadings and subsequent transfer to membrane were similar between the time points and between the lines. Therefore, highly dynamic changes in PSR1-YFP could not be accounted for by variation in protein loadings.

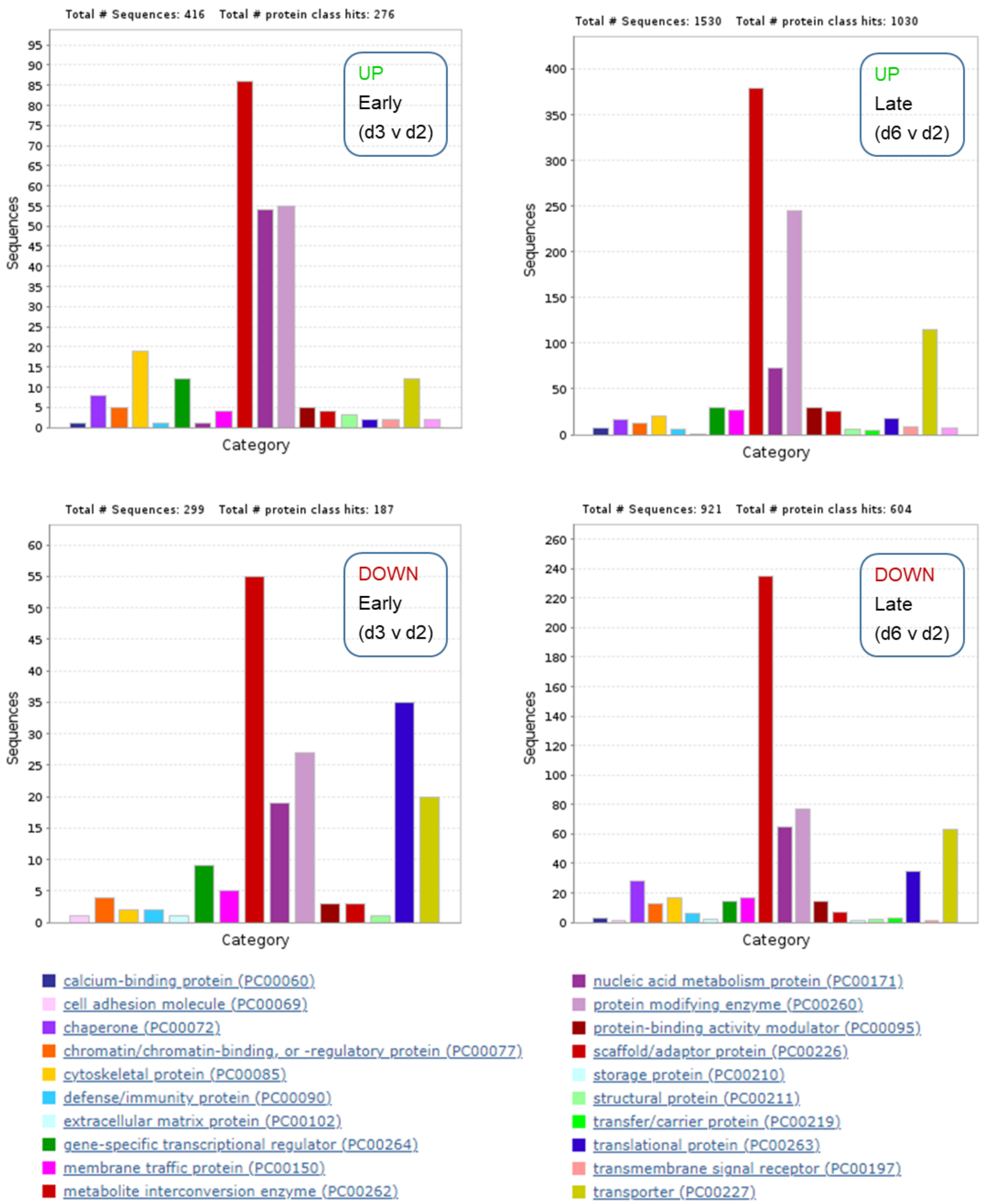

Supplementary Figure 9. **Gene function analysis of UVM4 during batch culture.** Analysis based on Panther gene ontology for protein class for early and late; up and down as labelled. Relative data was obtained (FC) using a 2-fold biological significance cut-off with a P-adj<0.05 statistical significance cut-off. This generated four sets of genes for early changes (d3 v. d2) and late (d6 v. d2) for both up and down (listed in **Data S1**). Protein classes are indicated in the legend and numbers falling into these classes in relation to the total are indicated at the top of each chart. Most of the changes at all stages, up or down were metabolite-interconversion enzyme genes, followed by protein modifying enzyme genes, nucleic acid metabolism proteins and transporters. Large early decreases are shown in translational protein and transporters, and a relatively large increase is shown in cytoskeletal protein genes.

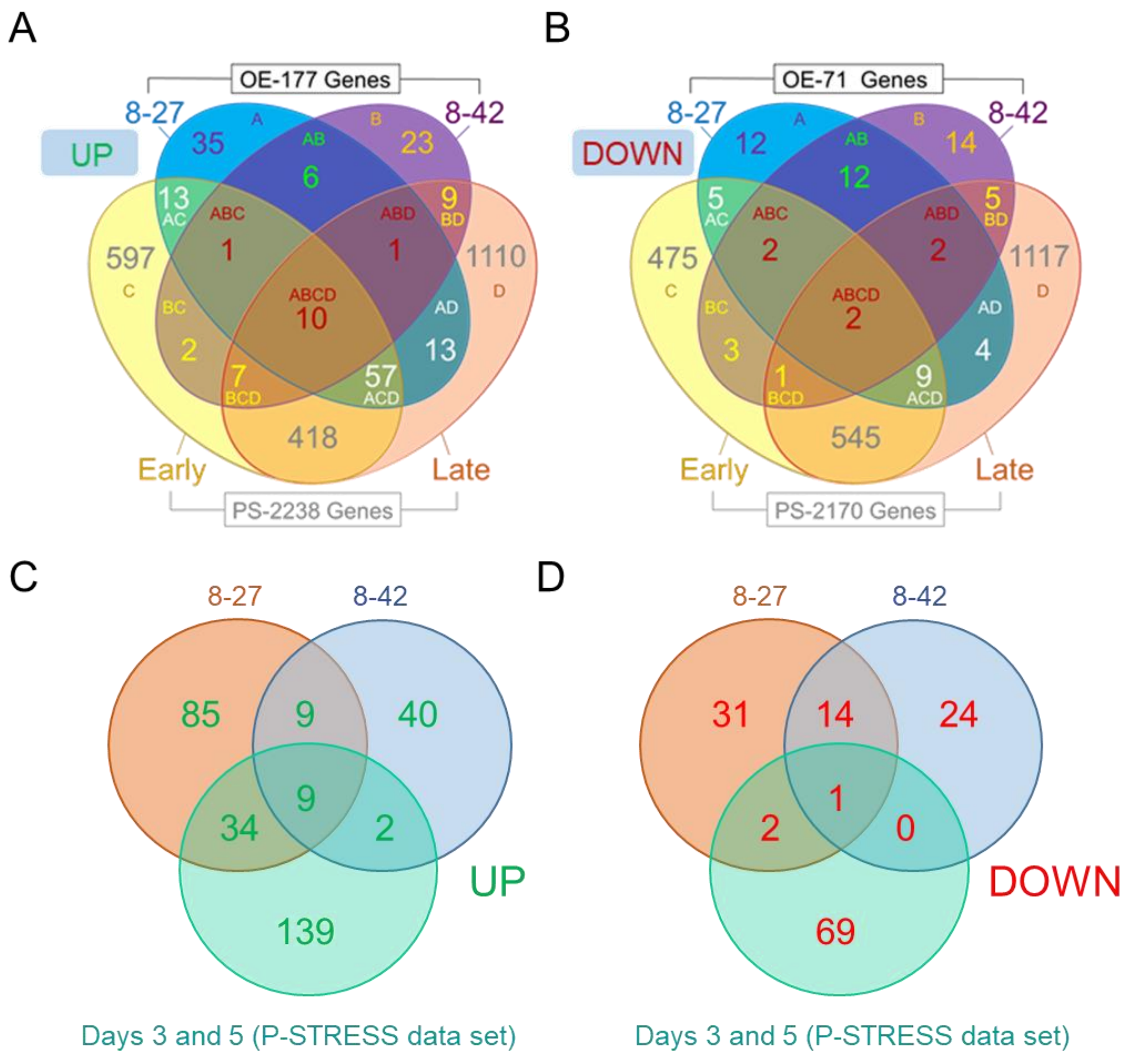

Supplementary Figure 10. **Co-regulated expression changes between two PSR1-OE lines (8-27 and 8-42) and the P-STRESS data showing direction of regulation (up or down)**. Co-regulation occurrences are shown for maximum (**A, C**) or minimum (**B, D**) FC values (≥2-fold FC relative to UVM4) for a given gene from the PSR1-OE lines in comparison with the P-STRESS dataset FC's. (**A, B**) Gene number per Venn sector is indicated and each sector is labelled as follows: A (8-27), B (8-42), C (P-STRESS d3 "EARLY") and D (P-STRESS d5 "LATE"). Here the P-STRESS FC cut-off was also ≥2-fold. (**C, D**) Similar analysis employing different cut-offs for the P-STRESS dataset FC's (**C**) ≥ 6-fold and (**D**) ≥ 25-fold to generate equivalent gene numbers as observed for OE-248 up (177) and down (71).

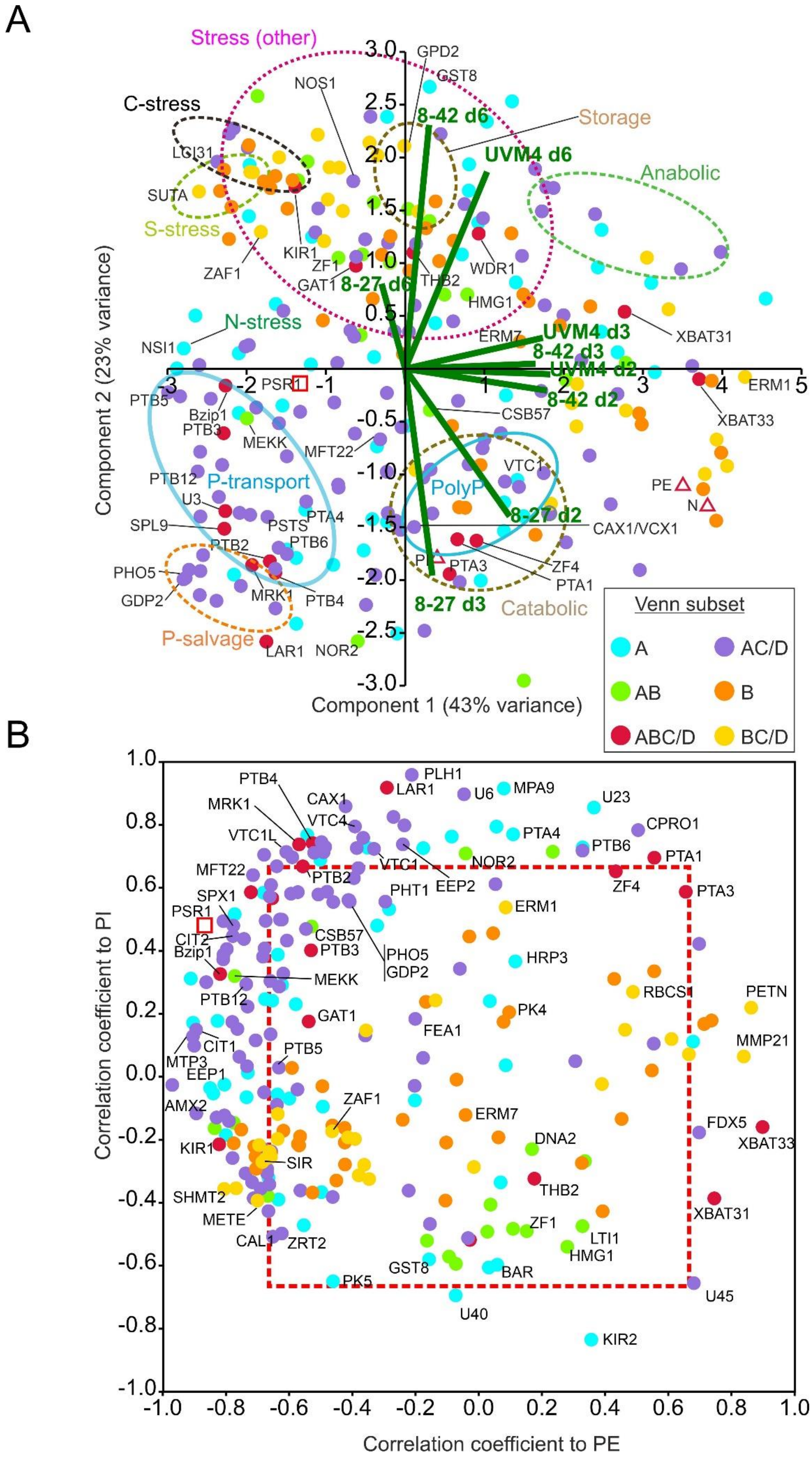
A
Stress (other)
C-stress
S-stress
N-stress
Storage
Anabolic
P-transport
PolyP
P-salvage
Catabolic
8-42 d6
UVM4 d6
8-27 d6
UVM4 d3
8-42 d3
UVM4 d2
8-42 d2
8-27 d2
8-27 d3
Component 1 (43% variance)
Component 2 (23% variance)
Venn subset
A
AC/D
AB
B
ABC/D
BC/D
B
Correlation coefficient to PE
Correlation coefficient to PI

Supplementary Figure 11. Temporal regulation of PSR1-OE gene expression. Patterns of gene expression focusing on temporal factors are shown for the OE-248 gene set. The charts are coded for the six Venn diagram sector subsets (inset) as described in **Figure 3C.** The data point for the full PSR1 gene mRNA (not specific to endogenous gene or transgene) is shown (□). (**A**) PCA analysis of normalized mean (n=3) RPKM and PE, PI and N measurements (Δ). Biplots (-) shown for three lines and timepoints. Clusters highlighting functional processes are encircled. (**B**) Plot of Pearson's correlation coefficients for RPKM data v. PI and v. PE for each OE-248 gene. Coefficients outside the boxed region (---) were significant for PI or PE ($P<0.05$). In all cases mean data was from n=3 culture replicates.

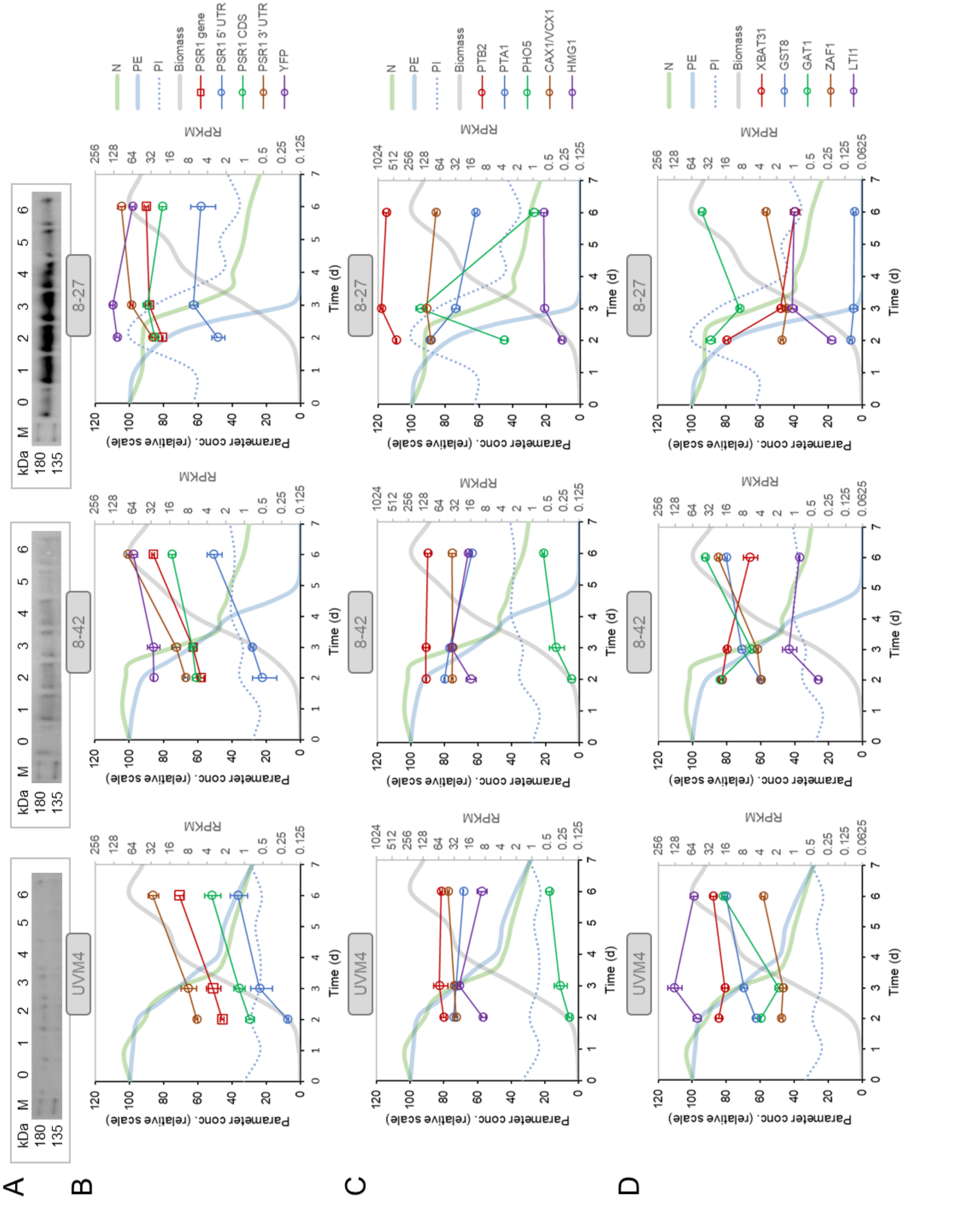

A
B
C
D
kDa
180
135
M
UVM4
8-42
8-27
Time (d)
Parameter conc. (relative scale)
RPKM
N
PE
PI
Biomass
PSR1 gene
PSR1 5' UTR
PSR1 CDS
PSR1 3' UTR
YFP
PTB2
PTA1
PHO5
CAX1/VCX1
HMG1
XBAT31
GST8
GAT1
ZAF1
LTI1

Supplementary Figure 12. mRNA expression levels of PSR1 and exemplar gene targets of PSR1-OE. Comparison of PSR1 mRNA and fusion protein expression levels with mRNA levels for target genes from Class I-IV. (**A**) Anti-YFP antibody western showing PSR1-YFP fusion protein with equal chlorophyll loadings (10 µg), where d0 refers to the starter culture prior to dilution. (**B)** mRNA levels determined by RPKM for PSR1 showing non-specific full mRNA ("gene") and CDS fragments, along with one transgene-specific YFP fragment and two endogenous gene-specific UTR fragments. Normalized PE, PI and N data shown for comparison. Error bars indicate SE, n=3 culture replicates. (**C**) RPKM data for Class I (PTA1, PTB2: PSR1 over-expression induced), Class II (PHO5: PSR1 over-expression induced), Class III (HMG1: PSR1 over-expression repressed). (**D**) RPKM data for Class I (XBAT31: PSR1 over-expression repressed), Class II (GST8: PSR1 over-expression repressed), Class III (ZAF1: PSR1 over-expression induced) and Class IV (LTI1: PSR1 over-expression repressed). Error bars indicate SE, n=3 culture replicates.

.

Supplementary Table 1. Primers used in cloning and screening of transformants

Primer	Sequence	Binding site
GG_RBCS2intron1_F1	tactGCCAGGTGAGTCGAC	Binds at: 874 -> 892; 6251 -> 6269
GG_RBCS2intron1_R1	GCTTCGAAATTCTTCAGCACC	Binds at: 1049 <- 1069; 6426 <- 6446
LC9_PSR1_screen274_rev	CGAGGGTTGCAGCCTTCC	Binds at: 1327 <- 1344
LC13_L2_PSADterm_for	gccaacgtgccacaccc	Binds at: 5453 -> 5469
LC14_L2_PSADterm_rev	gggtgtggcacgttggc	Binds at: 5453 <- 5469
LC15_AphVIII_rev	CAACCGGGATACCGACC	Binds at: 6475 <- 6491
LC16_HSP70_for	cgaacggtgacctccac	Binds at: 5927 -> 5943
LC18_mVenus_rev	CCAGCTCCACCAGGATG	Binds at: 4121 <- 4137
LC29_PSADprom_for	cgaccgagatggcttgc	Binds at: 804 -> 820
LC40_PSADprom-2_for	caggccagggacgattatgt	Binds at: 709 -> 728
LC42_PSR1_screen142_rev	GCTGTGCTGCGTCTTGATG	Binds at: 1194 <- 1212
LC43_PSADterm_rev	ctgtggctaattgaccgtgg	Binds at: 5337 <- 5356
LC45_mVenus_3'end_for	CATGGTGCTGCTGGAGTTC	Binds at: 5062 -> 5080
LC51_RT_RBCSint_for	cgtcgccgtttccatttg	Binds at: 1007 -> 1024; 6384 -> 6401
LC52_RT_3'UTR-PSAD_rev	cgatcccgtatcaatcagcg	Binds at: 5248 <- 5267
LC56_RT_PSR1-3'end_for	GACCCACTAGATGCGGATC	Binds at: 4002 -> 4020
LC56_RT_PSR1-3'end_rev	GATCCGCATCTAGTGGGTC	Binds at: 4002 <- 4020
LC57_RT_AphIII_screen79_for	GCTGGTGTTTATCGGCTTCG	Binds at: 6526 -> 6545
LC58_RT_AphIII_screen263_rev	GCTTCGGTGACCAACCAG	Binds at: 6693 <- 6710
LC61 RT_3'UTR-PSAD_2_rev	CTGCTGCCAGAACGCaag	Binds at: 5178 <- 5195
LC64_RT_PSR1-CDS992_for_pr3+	CTGAGCCCAGCATCTCTAGG	Binds at: 2299 -> 2318
LC65_RT_PSR1-CDS1184_rev_pr3+	CAGCTCCATTTGGAACAGCAG	Binds at: 2472 <- 2492
LC66_RT_PSR1-3'end_for	CGACCCACTAGATGCGGATC	Binds at: 4001 -> 4020
LC67 RT_VenusCDS_for	CACAAGTTCAGCGTGAGCG	Binds at: 4155 -> 4173
LC68 RT_VenusCDS_rev	CGTGCTGCTTCATGTGGTC	Binds at: 4308 <- 4326
LC71_RT_PSR1-CDS2009_for	GCAGGGTTTGCTCAATGACAG	Binds at: 3710 -> 3730
LC72_RT_PSR1-HA_rev	GGAACATCGTATGGGTACGC	Binds at: 3882 <- 3901; 3912 <- 3928; 3939 <- 3955
LC87_PSR1-ex1_for	GCATCTACTAGCACCGAGCG	Binds at: 1460 -> 1479
LC88_PSR1-ex2_rev	CGACGCCCATGAGCTTAAGG	Binds at: 1961 <- 1980
LC91 Venus-ex1_for	GACCACATGAAGCAGCACG	Binds at: 4308 -> 4326
LC92_Venus-ex2_rev	CCTTGATGCCGTTCTTCTGC	Binds at: 4879 <- 4898
LC95 PSR1-ex1_2_for	CCAGGCATTATGCATGGGC	Binds at: 1569 -> 1587
LC96 PSR1-ex2_2_rev	GTTCACCGCGTTGACGAAG	Binds at: 1904 <- 1922
LC101_RT_AphIII_rev	CAACACGAGGTACGGGAATCC	Binds at: 6644 <- 6664
LC102_qRT_Venus_for	GCACGACTTCTTCAAGAGCGCC	Binds at: 4322 -> 4343
LC103_qRT_Venus_rev	CCTTCAGCTCGATGCGGTTCAC	Binds at: 4769 <- 4790
LC104 PSR1-ex1-pr3+_for	CGCTGGGTGGATATGGACTC	Binds at: 1246 -> 1265
LC105 PSR1-ex1-pr3+_rev	GGCCAGCATGTACTCTGAGG	Binds at: 1420 <- 1439
LC106 PSR1-ex3-pr3+_for	AGCCTGATATGTCGCAGCAA	Binds at: 3652 -> 3671
LC107 PSR1-ex3-pr3+_rev	GTCCCCGAAATCACCGAAGT	Binds at: 3787 <- 3806
PM829_GGAphVIII_F1	ttGAAGACatAATGgacgatgcgttgcgtg	Binds at: 6447 -> 6466
PM830_GGAphVIII_R1	ttGAAGACatAAGCtcagaagaactcgtccaacagc	Binds at: 7230 <- 7255
PM831_AphVIII_F2	CGGGAGTTGTTTGTCAAGGT	Binds at: 6556 -> 6575
PSR1_BpiI_CDSinclStop_rev	TTGAAGACATAAGCCTATGGCTCCACTCGCTGCC	Binds at: 4030 <- 4046
PSR1_BpiI_CDSns_rev	TTGAAGACATCGAACTTGGCTCCACTCGCTGCC	Binds at: 4030 <- 4052
PSR1_BpiI_for	TTGAAGACATAATGGACAAAGCTGAACGCGCTGCT	Binds at: 1070 -> 1094

Supplementary Table 2. Plasmids used in the construction of the pLC8 construct

Name	Description	Selection	Backbone plasmid	Creator	SnapGene file
pAGM1287	Level 0 backbone vector	Spec			pAGM1287.dna
pICH41308	Level 0 backbone vector	Spec			pICH41308.dna
LC10.3/4	PSR1-HAns_L0 (gDNA of transcription factor PSR1 HA-tagged no stop codon in pAGM1287)	Spec	pAGM1287	Lili Chu	pAGM1287.dna
PM_L1_TU4_paroR	TU paromomycin resistance cassette	Amp/Carb	pICH47781	Payam Mehrshahi (Cambridge)	PM_L1_TU4_paroR_AphVIII_pICH47781.dna
L1_pLC8	PSADprom-5'UTR – RBCS2intron – PSR1-HAtagns – C-tagVenus – PSADterm	Amp/Carb	pICH47772	Lili Chu	L1_pLC8_pICH47772.dna
L2_pLC8	L1_pLC8 + ParoR	Kan/Paro	pAGM4673	Lili Chu	L2_pLC8.dna
pFJN3	Level 0 RBCS2intron	Spec		Cambridge	pFJN3.dna
pFJN40	Level 0 Venus	Spec		Cambridge	pFJN40.dna
pFJN43	Level 0 PSAD promoter incl. 5'UTR	Spec		Cambridge	pFJN43.dna
pFJN44	Level 0 PSAD promoter without 5'UTR	Spec		Cambridge	pFJN44.dna
pFJN45	Level 0 PSAD 3'UTR terminator	Spec		Cambridge	pFJN45.dna
